# Body composition changes in the postpartum: a systematic review on measurements and predictors

**DOI:** 10.1186/s12884-026-09393-7

**Published:** 2026-06-06

**Authors:** Malith Kumarasinghe, Andrew P Hills, Kiran DK Ahuja

**Affiliations:** School of Health Sciences, UTAS Health, University of Tasmania (UTAS), The Shed, 80, Cimitiere Street, Inveresk, Launceston, TAS 7250 Australia

**Keywords:** Postpartum, Body composition, Factors, Systematic review

## Abstract

**Introduction:**

This systematic review investigated changes in maternal body composition and specific anthropometric indices during the postpartum period, and the factors influencing these changes.

**Methods:**

Longitudinal observational studies with at least two measurements of postpartum maternal body composition and anthropometry were included. Studies that assessed only BMI or included a single postpartum measurement without a corresponding preconception measure were excluded. Medline/PubMed, Scopus, EMBASE, CINAHL, Web of Science, and ProQuest databases were searched for studies published up to 30th September 2025. Conference proceedings or studies not published in English were excluded.

The risk of bias was assessed using the *Assessing risk of bias and confounding in observational studies of interventions or exposures tool* from the Agency for Healthcare Research and Quality. Data on the changes in body composition and associated factors were summarised and presented in graphical and tabular formats. The review was reported according to the Preferred Reporting Items for Systematic Reviews and Meta-analyses guidelines.

**Results:**

Of 1,273 abstracts screened, 86 studies were retained for data extraction. Sixty-one studies assessed changes in body composition and anthropometry, while 44 studies examined the association between various factors and body composition outcomes. Overall, 73 body composition and anthropometric indices were reported, with fat mass ratio/percentage being the most frequently used measure. Waist circumference showed a substantial reduction during the first month of the postpartum. The timing of initial measurements, length of follow-up, and techniques varied widely; 59% of studies concluded follow-up at or before 9 months postpartum. Techniques used to assess body composition ranged from skinfold thickness and circumference measurements to bioelectrical impedance analysis and dual-energy X-ray absorptiometry.

**Conclusions:**

This systematic review underscores the complexity and variability of postpartum body composition changes. Given the association between postpartum adiposity and adverse long-term maternal health outcomes, integrating body composition and anthropometric assessments alongside BMI is essential. Extending follow-up beyond one year postpartum may further improve understanding and management of postpartum adiposity in clinical practice.

**Supplementary Information:**

The online version contains supplementary material available at 10.1186/s12884-026-09393-7.

## Background

 Progressive changes in maternal body composition and specific anthropometry occur during pregnancy to facilitate the optimal foetal development and are reflected as variations in fat mass (FM) and fat-free mass (FFM). A return to pre-pregnancy body composition following delivery is important to facilitate healthy transition from pregnancy to the non-pregnancy state [1]. Conversely, suboptimal postpartum maternal body composition and anthropometry may result in both short-and long-term health consequences, including persistently elevated visceral FM [2], and an increased risk of cardiovascular diseases, and metabolic syndrome [3].

A range of maternal sociodemographic, lifestyle, and clinical factors influence postpartum changes in body composition and anthropometry. These include commonly considered 2009 Institute of Medicine (IOM) guidelines for gestational weight gain such as age, diet, breastfeeding gestational diabetes, and pregnancy-induced hypertension [4–9]. While the relationship of primary drivers such as excessive gestational weight gain, breastfeeding, diet, and physical activity and overall body weight or BMI have been widely appraised [10–12], evidence related to specific body composition indices and anthropometric measure remain poorly synthesized [13]. The lack of a systematic evaluation limits the ability to differentiate changes in adiposity from overall body weight and makes it difficult to identify gaps in the literature or inform clinical guidelines. Consequently, the limited evidence on factors influencing postpartum body composition may hinder efforts to optimise maternal health outcomes [14]. Thus, the objective of this systematic review was to investigate changes in maternal body composition and specific anthropometric measures during the postpartum period, and the factors influencing these changes.

## Materials & methods

### Eligibility criteria

Longitudinal, cohort, and case-control studies published in peer-reviewed journals, or thesis/dissertation repositories, on or before 30th September 2025 were included (Supplementary Table 1). This review considered body composition changes up to two years postpartum to capture the influence of maternal breastfeeding, consistent with the World Health Organization’s breastfeeding recommendations [15]. Studies assessing the efficacy of intervention or treatment designed to modify body composition in the postpartum period [16, 17] were excluded as the aim was to describe the ‘natural’ changes in postpartum body composition and specific anthropometric measures, and the factors influencing this change. The review protocol was prospectively registered in PROSPERO (ID: CRD42024556305).

### Information sources

Medline/PubMed, Scopus, EMBASE, CINAHL, Web of Science, and ProQuest were searched.

### Search strategy

A combination of Medical Subject Headings (MeSH) and relevant keywords relating to the postpartum period and body composition were used (Supplementary Table 2; Supplementary Sheet 1). Terms related to potential determinants were not explicitly included in the search strategy to maximise sensitivity and ensure that relevant documents were not missed.

### Study selection

Search results from individual databases were imported into EndNote^®^ (Version 21, Clarivate, United Kingdom), to remove duplicates, before exporting them to Covidence^®^ (Veritas Health Innovation, Australia) for screening. Title and abstract screening were conducted independently by two reviewers (MK and KDKA). Full texts of potentially eligible studies were retrieved. One reviewer (MK) performed the full-text screening, while the second reviewer (KDKA) independently reviewed studies where eligibility was uncertain or where disagreements arose during the initial screening. Reasons for exclusion were recorded and verified by both authors (Supplementary Sheet 2, 3 & 4).

### Data extraction and management

Two authors independently extracted relevant data from all the eligible studies using a purpose-built Excel template (Supplementary Sheet 2, 3 & 4).

### Assessment of risk of bias

The risk of bias was assessed using the Agency for Healthcare Research and Quality’s *Assessing Risk of Bias and Confounding in Observational Studies of Interventions or Exposures* tool [18]. This tool assesses 13 criteria across six domains: selection bias, performance bias, detection bias, attrition bias, selective outcome reporting, and confounding [18].

### Data synthesis

Eligible studies were summarised based on body composition and specific anthropometric indices (e.g., FM, and FFM), timing of initial postpartum measurements (within 7 days, between 7 and 42 days, and > 42 days postpartum), and factors assessed for association with these outcomes (e.g., mother’s age, education, and breastfeeding). Data were also stratified by preconception BMI (< 18.5 kg/m^2^, 18.5 kg/m^2^–24.9 kg/m^2,^ and ≥ 25 kg/m^2^), to explore patterns of change in body composition and anthropometry. Where studies reported subgroup specific data rather than total population estimates, each sub-group was treated as a separate dataset. Graphs were generated using GraphPad Prism (Version 10, GraphPad Software, USA). Results for the top five commonly reported body composition and anthropometry measures (calculated as change), as well as factors assessed for their association were visualised. A minor discrepancy exists between the study counts reported in the text and figures: figures include only studies with two or more postpartum measurements, while the text also includes studies with preconception baseline measurement.

## Results

From 1,273 abstracts screened, 86 studies published between 1983, and 30 September 2025 met the eligibility criteria. Of these, 61 studies assessed changes in body composition and specific anthropometric measures, and 44 examined the association between maternal factors and these changes (Supplementary Fig. 1; Supplementary Sheet 2, 3 & 4). Overall, detection and selection bias were generally low. All studies used valid measurement methods, although one study applied inconsistent inclusion criteria with postpartum participants selected based on breastfeeding status and maternal/infant health factors, while controls were selected based on BMI, weight stability, and general health status [19]. Attrition bias was the most prominent concern: 40 studies reported > 20% loss to follow-up [4, 20–58], yet only seven studies assessed its potential impact [20–26]. Attrition could not be determined in five studies [59–63]. Reporting bias was identified in seven studies [27, 64–69]. Performance bias and comparative confounding were largely not applicable due to the single‑arm longitudinal designs.

Only 33 of the 86 studies consisted of ≥ 50 participants [20–22, 24, 26, 29–31, 35, 36, 38, 41, 43, 44, 50, 52, 58–64, 67, 70–78], and 18 of these had sample size exceeding 100 participants [20–22, 24, 26, 29, 30, 35, 36, 38, 52, 59, 62, 63, 75–78]. Across all studies, 73 unique body composition and anthropometric indices were reported.

**Fig. 1:**
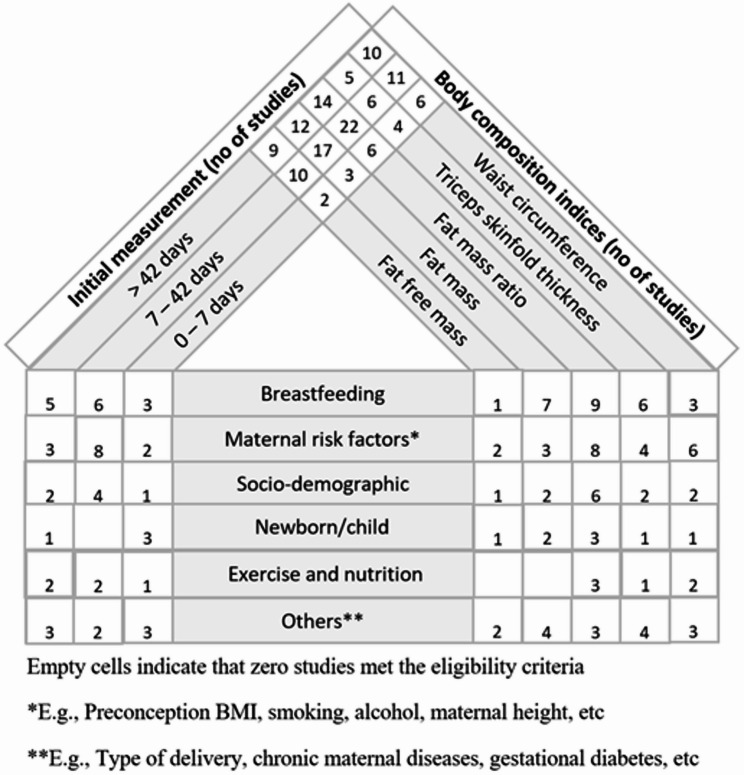
Number of eligible studies investigating changes in body composition and/or anthropometric measures and associated factors, separated by timing of initial postpartum measurement

The five most common indices with at least two measurements in the postpartum (Fig. 1) were fat mass ratio (FMR; 42 studies), FM (32 studies), waist circumference (WC; 27 studies), FFM (21 studies), and triceps skinfold thickness (TC; 15 studies). The initial measurement for most studies was within 42 days postpartum (*n* = 62). Follow up duration ranged from 30 days postpartum [59] to up to 2 years [41, 60, 75, 79–81].

Of the 21 studies assessing FFM [2, 4, 19, 20, 22, 27, 31, 41, 44–47, 49, 65, 67, 71, 81–85], only seven reported sample sizes ≥ 50 [20, 22, 31, 41, 44, 67, 71]. All but two studies [2, 44] measured FFM after 7 days postpartum. Additionally, just over half (12/21) obtained initial FFM measurement within 42 days postpartum [2, 19, 22, 27, 44–46, 49, 65, 67, 83, 86], and only seven studies followed participants for at least one year [4, 27, 41, 46, 71, 81, 83].

Fifteen studies assessed TC [20, 24, 32, 44, 48, 52, 59, 65, 66, 68, 80, 81, 87–89] with 10 initiating measurement within 42 days postpartum [32, 44, 48, 52, 59, 65, 68, 80, 87, 88]. Among the 27 studies measuring WC [19, 21, 23, 24, 26, 30, 32, 34–37, 51, 54, 59, 60, 62, 63, 65, 69, 75–78, 80, 85, 89, 90], 17 initiated follow-up within 42 days postpartum [19, 21, 23, 30, 32, 34, 36, 37, 51, 54, 59, 63, 65, 69, 77, 78, 80] and13 studies followed participants for one year [23, 26, 30, 32, 34, 35, 51, 54, 60, 62, 75, 76, 80].

Overall, 46 factors (Fig. 1) were assessed for their association with changes in body composition and anthropometric measures. The most frequently assessed factors were breastfeeding (16 studies), maternal risk factors (16 studies), socio-demographic characteristics (9 studies), exercise and nutrition (9 studies). Newborn/child related factors were assessed in four studies. Most other factors were evaluated by only a single study. For example, with exception of breastfeeding, maternal weight indices, age, and income, all other factors related to FMR were assessed by individual studies only. Notably, the largest study assessing breastfeeding and postpartum body composition change included 100 participants and was published in 1989 [38]. None of the studies investigating the association of breastfeeding or weight-related factors with body composition change followed participants beyond one year postpartum. Only one study exploring socio-demographic factors (occupation) continued until 13 months [81].

Most studies used multiple assessment methods, including body circumference, skinfold thickness, bioelectrical impedance analysis (BIA), and dual-energy X-ray absorptiometry (DXA). Similarly, a wide range of measurements were reported, including 14 different circumference indicators (e.g., waist, hip, mid-arm circumferences), 16 skinfold thickness measurements or their composite scores (e.g., triceps, thigh, the sum of four skinfolds), 23 regional fat/lean mass measures (e.g., arm fat mass, android fat mass, visceral fat mass). Measures of fluid balance and whole-body fat, lean, or cell mass were also reported.

### Change in postpartum body composition

**Fig. 2:**
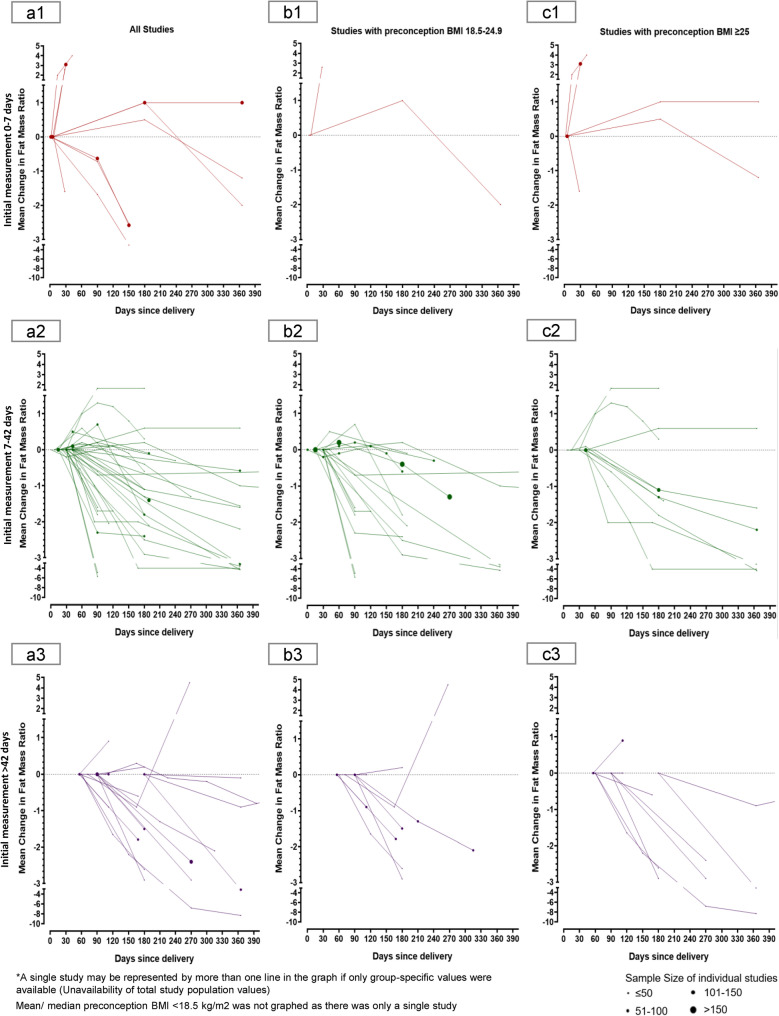
Change in postpartum fat mass ratio by timing of initial measurement and preconception BMI

Two distinct patterns of FMR change emerged, irrespective of the day of initial postpartum measurement (Fig. 2a1-a3). Majority of studies (30/42) showed a rapid FMR decline of up to 4% within the first six months postpartum, followed by a slower loss or plateau through one year and beyond [4, 20, 21, 24, 27, 30–32, 34, 42–44, 48, 55, 56, 58, 60, 64, 65, 71, 72, 80, 83–85, 87–89, 91, 92]. In contrast, 18 of 42 studies observed an initial FMR increase of 1–2% during the first 3 months, followed by a modest reduction of approximately 2% or less [2, 23, 24, 30, 31, 46, 49, 54, 56, 59, 61, 64, 65, 67, 70, 79, 81, 93] (Fig. 2a1 & a2). While examining the change from preconception to postpartum, only one of five studies reported a 0.9% decrease in FMR [82], whereas all other demonstrated increases of at least 2% [46, 93–95].

Ten studies reported an increase in FM during the first 2–3 months postpartum, although this initial rise was generally small (typically < 1 kg) [2, 20, 22, 24, 30, 31, 65, 67, 79, 93] (Supplementary Fig. 2a1-a3). Regardless of this initial rise, most studies (28 out of 32) demonstrated an overall decline in FM across the postpartum period [4, 19, 20, 22, 24, 27, 30, 31, 34, 41, 44–46, 49, 65, 67, 69, 71, 73, 79, 81, 83–87, 93, 96]. From preconception to postpartum, only one of six studies observed a 2.3 kg reduction in FM [82] whereas other studies reported increases of at least 2 kg [46, 47, 50, 86, 97].

**Fig. 3:**
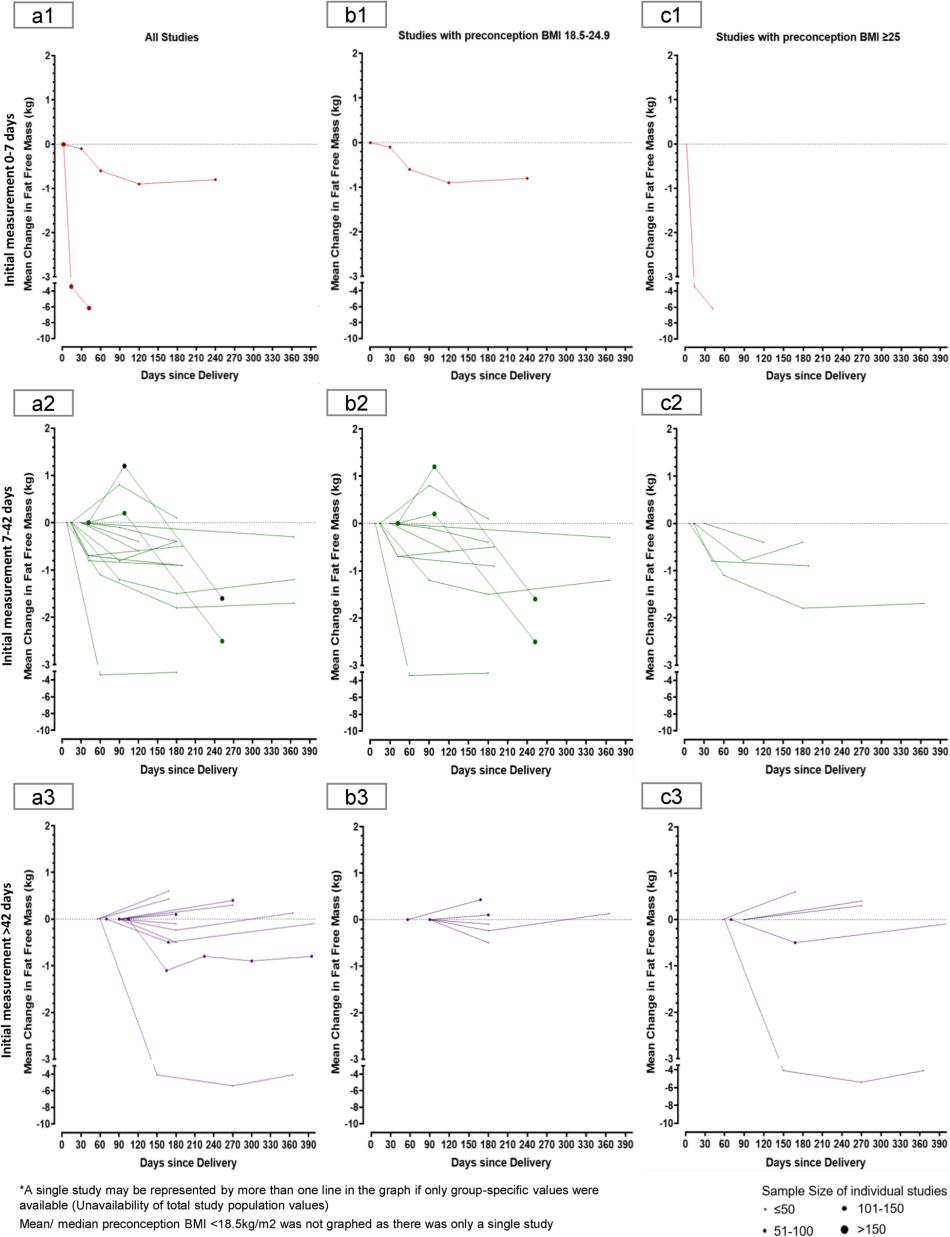
Change in postpartum fat-free mass by timing of initial measurement and preconception BMI

FFM followed a similar pattern to FM during the first 42 days postpartum. However, only seven of the 21 studies reported a reduction greater than 1 kg [2, 4, 22, 46, 65, 83, 86] (Fig. 3a1-a3). Beyond this period, changes in FFM were generally modest, with fluctuations typically < 1 kg after three to four months postpartum.

**Fig. 4:**
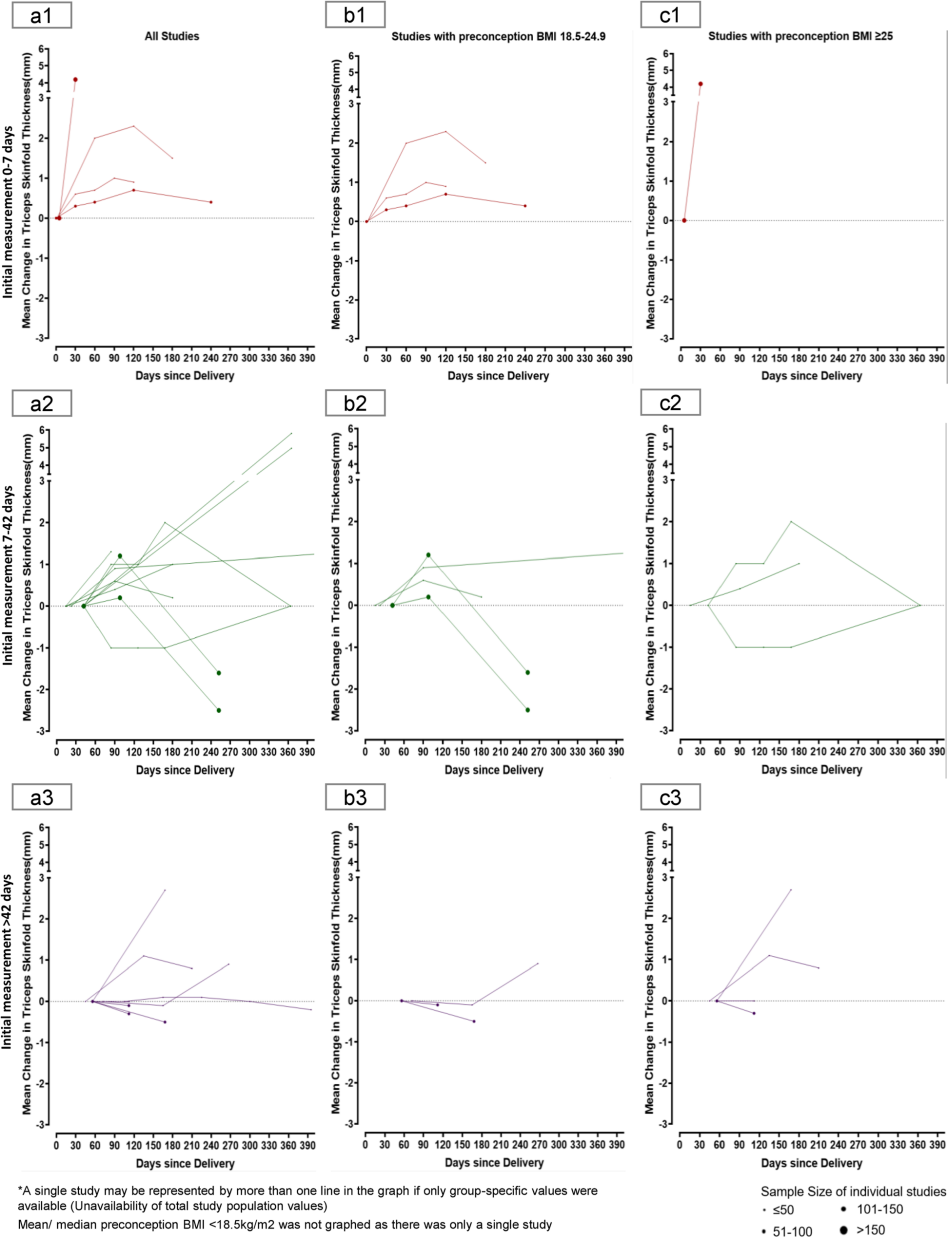
Change in postpartum triceps skinfold thickness by timing of initial measurement and preconception BMI

TC appeared to increase in varying degrees during the first 2–3 months postpartum. In 10 [20, 22, 24, 44, 65, 66, 68, 80, 87, 88] of the15 studies, the increase was < 2 mm (Fig. 4a1-a3) [20, 24, 32, 44, 48, 52, 59, 65, 66, 68, 80, 81, 87–89]. Following this initial increase, two distinct trajectories emerged: five studies reported decreases towards the baseline or slightly below [22, 44, 48, 65, 66, 87], while three studies showed continued increases [65, 80, 88].

Twenty-two of 27 studies on demonstrated at least 1 cm reduction in WC during the postpartum period [21, 23, 26, 30, 32, 34, 36, 37, 51, 54, 59, 62, 63, 65, 69, 75–78, 85, 89, 90]. The largest decreases were observed in studies with initial assessment within the first seven days after delivery – where 5 out of 6 studies reported reductions > 4 cm [23, 37, 54, 59, 63, 69]. Studies initiating measurements between 7 and 42 days postpartum reported reductions over 2 cm (5 out of 11 studies) [19, 21, 30, 32, 34, 36, 51, 65, 77, 78, 80]. Among studies beginning after 42 days, seven of ten studies reported decreases > 1 cm) [24, 26, 35, 60, 62, 75, 76, 85, 89, 90] (Supplementary Fig. 3a1-a3).

### Factors associated with change in postpartum body composition and anthropometry

#### Influence of maternal preconception and postpartum weight-related factors

FMR did not substantially deviate from the overall change in postpartum following the grouping by pre-conception BMI of 18.5 to 24.9 kg/m^2^ (Fig. 2b1-b3). However, among women with preconception BMI > 25 kg/m^2^, most studies (3/5 studies) showed that FMR did not decline after the early increase when measurements were initiated within first postpartum week [2, 54, 59] (Fig. 2: c1).

The overall trajectory of FM persisted regardless of the preconception BMI status (Supplementary Fig. 2: b1-b3 & c1-c3). Moreover, the course of FFM or TC also did not vary with preconception BMI (Fig. 3b1-b3 & c1-c3; Fig. 4b1-b3 & c1-c3). Few studies (*n* = 4) reporting an increase in WC in the postpartum period were conducted among women classified as overweight or obese prior to pregnancy [24, 30, 60, 77] (Supplementary Fig. 3: c2-c3). 

**Fig. 5:**
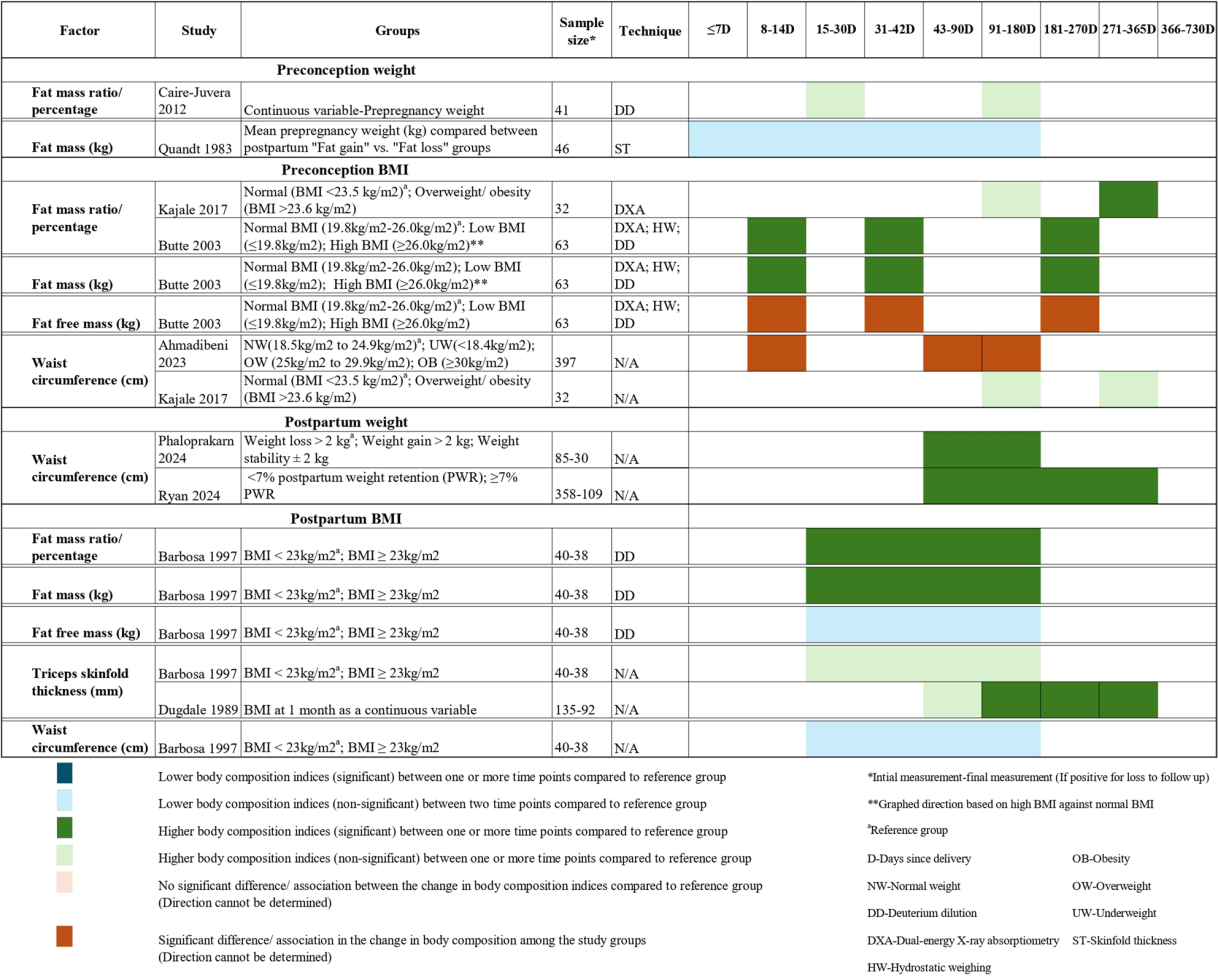
Association between maternal weight-related factors and changes in selected postpartum body composition and anthropometric measures

Nine studies investigated the association between weight-related factors, namely preconception BMI, weight, postpartum weight, and postpartum BMI changes in FM, FMR, FFM, TC, and WC [26, 36, 38, 54, 63, 65, 67, 92, 98] (Fig. 5). However, only four studies were published after 2012 [26, 36, 54, 63]. Deuterium dilution, an isotope-based method for estimating total body water and deriving FM, and FFM, was the most commonly used technique in these analyses. No significant association was observed between preconception weight and either FM or FMR [92, 98]. A higher postpartum BMI was associated with FM, FMR, and TC [38, 65], while higher postpartum weight was associated with higher WC [26, 36]. Additionally, higher preconception BMI was consistently associated with FM, FMR, and WC [54, 67].

#### Sociodemographic factors

**Fig. 6:**
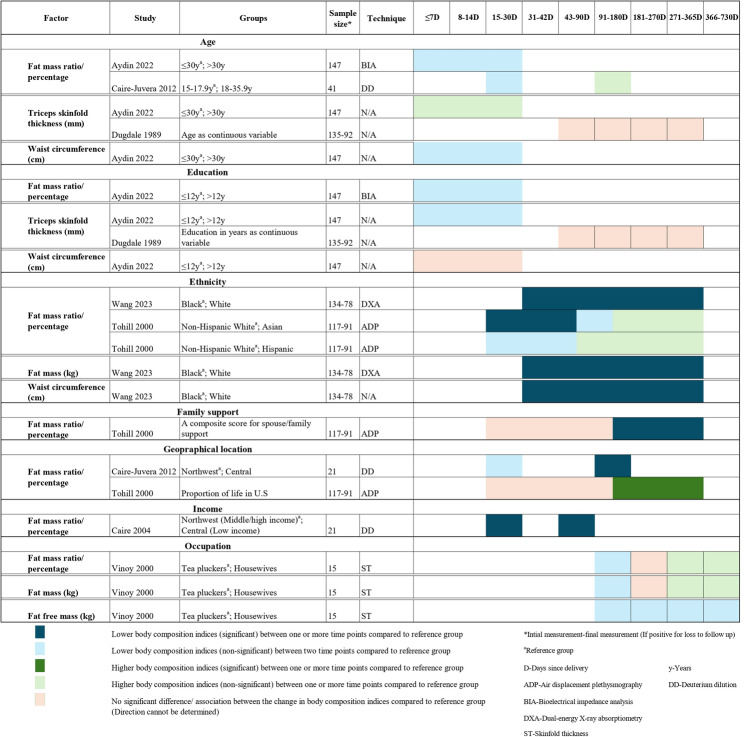
Association between sociodemographic factors and changes in selected postpartum body composition and anthropometric measures

A range of sociodemographic factors were examined, including age, education, ethnicity, family support, geographical location, income and occupation (Fig. 6) [29, 30]. White ethnicity was associated with higher body composition and anthropometric indices compared to Black ethnicity from the two-month to one year postpartum [30]. In contrast, among Asian and Hispanic ethnicities, the association with FMR shifted over time from lower to higher values relative to non-Hispanic White women in early (2–3 months) postpartum [29]. However, maternal age, education, and occupation were not significantly associated with the changes in postpartum body composition and anthropometric measures [38, 59, 81, 92] (Fig. 6). The influence of place of residence and family support on FMR was inconsistent, with findings ranging from non-significant to significant beyond the first 2 to 3 months [29, 92] (Fig. 6).

#### Influence of breastfeeding-related factors on changes in postpartum body composition and anthropometry

**Fig. 7:**
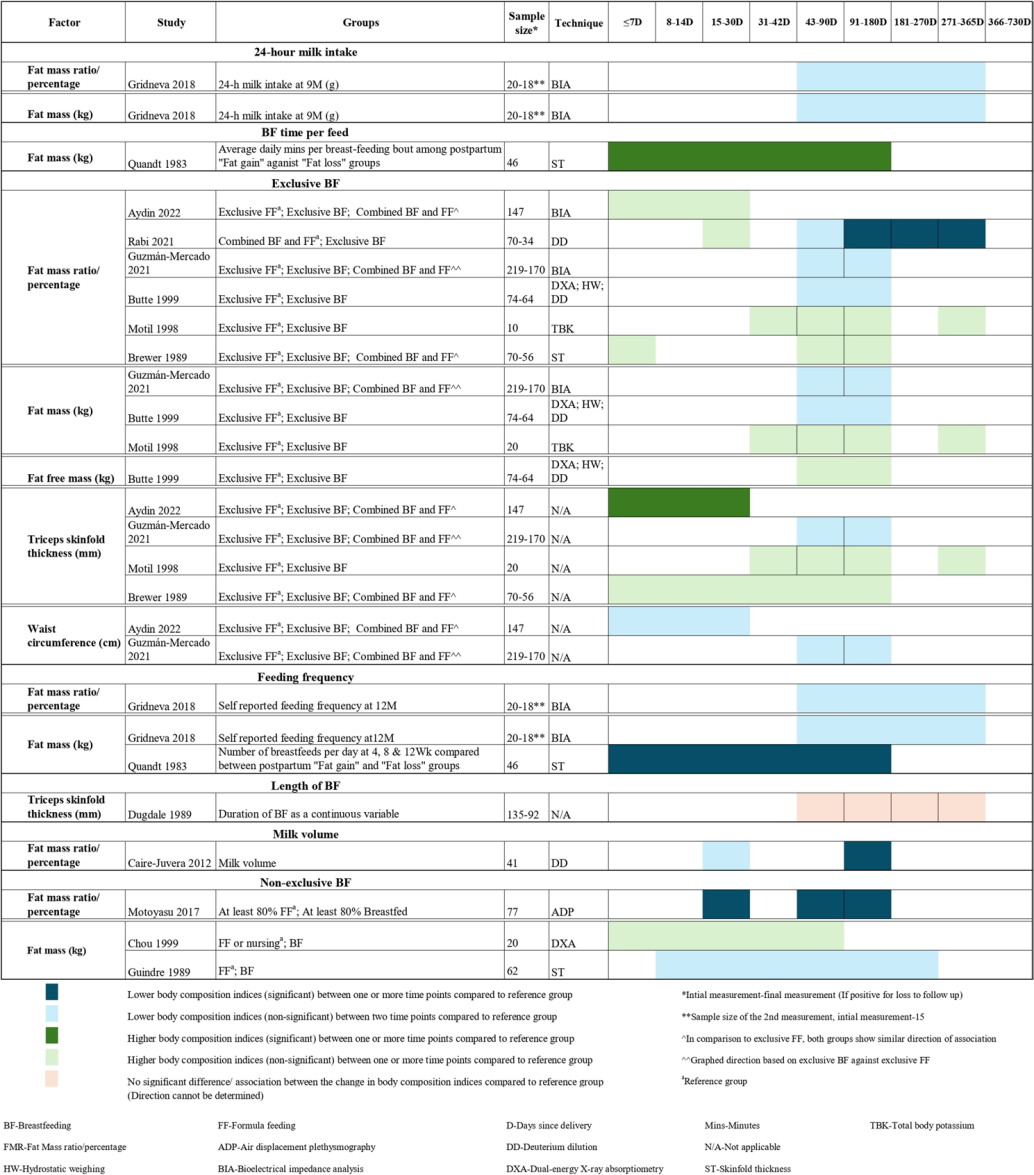
Association between breastfeeding-related factors and changes in selected postpartum body composition and anthropometric measures

Several breastfeeding-related factors were examined in relation to changes in postpartum body composition and anthropometry measures, including as 24-hour milk intake, feeding duration, exclusivity of breastfeeding, feeding frequency, and overall duration and volume of breastfeeding (Fig. 7). Only two longitudinal studies reported a sample size exceeding 100 [24, 38], one of which was conducted over three decades ago [38] (Fig. 7). within the 42 days postpartum, exclusive breastfeeding mothers generally exhibited higher FM, FMR, and TC alongside a lower WC compared to formula feeding mothers, however, most of these differences were not statistically significant [55, 58, 59, 87]. Beyond 42 days postpartum, results were inconsistent. Some studies reported lower FM, FMR, and TC among exclusive breastfeeding mother, whereas others showed a continuation of the relatively higher body composition indices [24, 31, 55, 58, 59, 87] (Fig. 7). In contrast, the lower FM and FMR were reported with larger milk volumes, increased feeding frequency, and higher 24-hour milk intake up to 6 to 12 months postpartum [4, 92, 98] (Fig. 7).

#### Influence of selected factors on the change in postpartum fat mass ratio

**Fig. 8:**
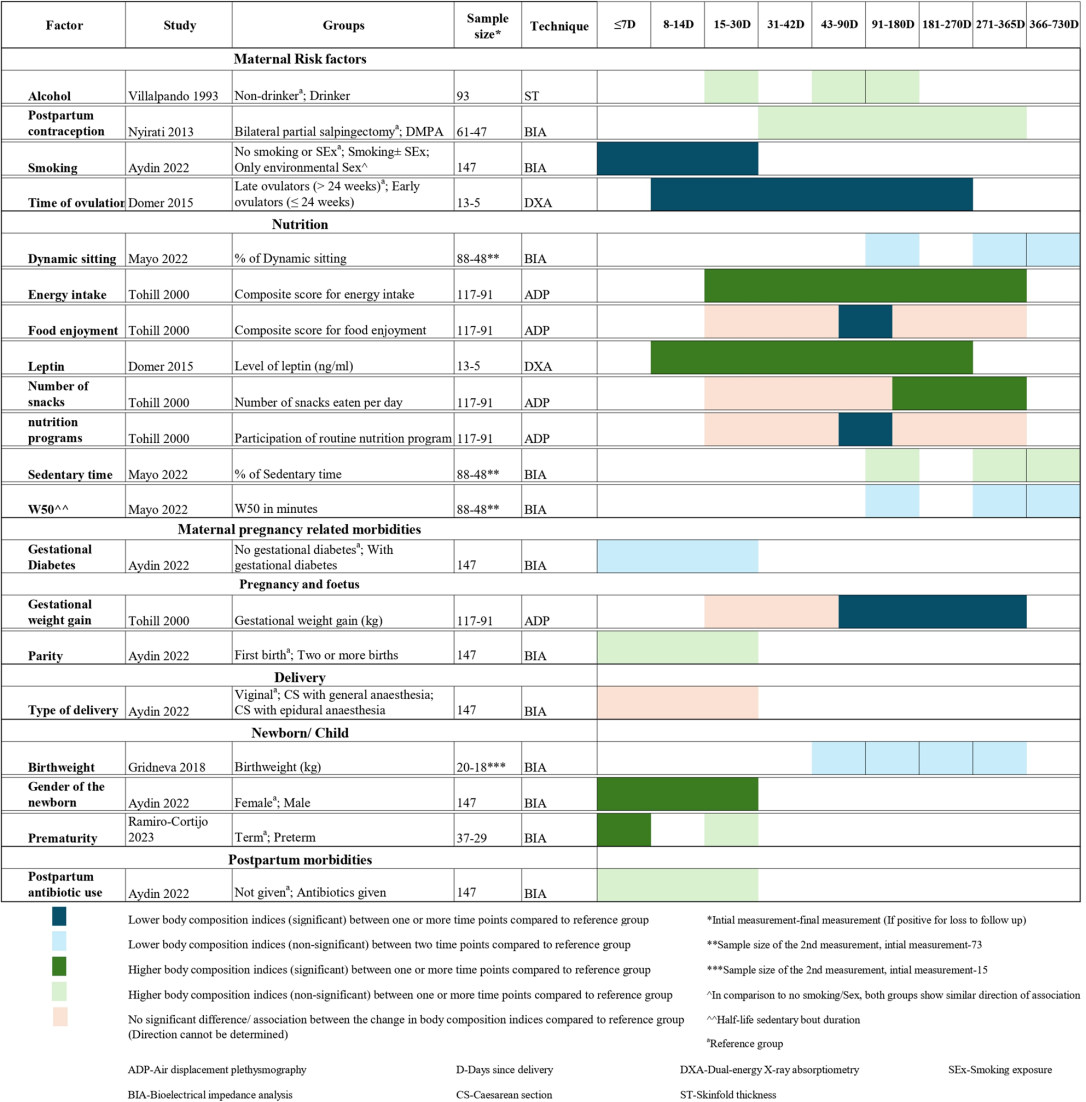
Association between selected factors and changes in postpartum fat mass ratio/ percentage

A range of maternal and neonatal factors were assessed for their association with changes in postpartum FMR, including maternal clinical risk factors, dietary behaviour, and pregnancy-related morbidities (Fig. 8). BIA was the commonly used technique in these studies [4, 32, 59, 60]. Higher leptin levels, increased energy intake, and male infant sex were associated with a significantly higher FMR during the postpartum period [29, 57, 59]. Additionally, food enjoyment and participation in routine nutritional programs were linked to a higher FMR between 90 and 180 days postpartum, although these associations did not persist through the entire postpartum period [29]. Conversely, smoking and early resumption of ovulation were associated with lower postpartum FMR [57, 59]. An increased number of daily snacks consumed was linked with a greater decline in FMR in the latter half of the first postpartum year; however, this association was not significant when considering the overall change in FMR [29]. Conversely, higher gestational weight gain was associated with a significant reduction in overall postpartum FMR in the same study [29] (Fig. 8).

Evidence on the association of HIV status on postpartum body composition and anthropometry was limited. Only TC demonstrated a reduction among women with HIV between the second to ninth months postpartum [20, 22] (Supplementary Fig. 4). No consistent associations were observed between HIV status and FM, FMR, and FFM [20, 22, 52] (Supplementary Fig. 4).

## Discussion

### Aim of the review

This review aimed to synthesise evidence on postpartum changes in body composition and specific anthropometry markers, and their association with maternal factors. Understanding natural trajectories of body composition; and existing knowledge gaps is essential for informing clinical guidelines and supporting optimal maternal outcomes in subsequent pregnancies.

Although our methodology was robust, the included studies showed substantial heterogeneity in measurement timing, techniques of body composition and anthropometric measurements, and data reporting. While 44 studies examined 46 maternal most were assessed in only one or two studies (e.g., parity 1 study; mode of delivery 1 study; ethnicity 2 studies) limiting meaningful comparison and synthesis. Similarly, variability in data presentation (e.g., breastfeeding type 6 studies but with different combinations of exclusivity of breastfeeding/ formula feeding) further complicated the interpretation. Attrition was also a key limitation, with 40 out of 86 studies reporting loss to follow up rates of ≥ 20% [4, 20–58]. Post hoc analyses showed similar attrition across identified fat mass ratio (FMR) trajectories (rapid reduction: 56.7%; transient increase: 55.5%), suggesting comparable distribution between patterns. However, attrition in postpartum studies is unlikely to be random [99, 100], as women experiencing adverse outcomes may be more likely to withdraw. This may bias findings toward more favourable trends, warranting cautious interpretation of commonly observed fat mass reductions by 6–12 months postpartum.

Overall, the strength of evidence for specific maternal behavioural and sociodemographic factors remains low-to-moderate due to heterogeneity and limited data per factor. While the natural trajectories are well-mapped, more standardized, low-attrition longitudinal designs are required to strengthen the certainty of evidence. Accordingly, we highlight key areas requiring further investigation.

### Time of measurement

Determining the timing, duration and frequency of body composition measurements is critical for accurately capturing postpartum changes. Although early measurement (soon after delivery) is ideal, it is often impractical during the immediate (first 24 h) and early postpartum period (days 2–7/14) [99]. In the first 1–2 weeks postpartum, rapid diuresis from pregnancy-related fluid loss distorts FM and FFM estimates [2, 44, 100–104], with the initial exponential decline slowing to a more gradual, linear pattern by the end of this period [102].

In our review, the rapid decrease of up to 4% FMR in the first six postpartum months may partly reflect inconsistencies in early measurement (Fig. 2). Initiating assessments after the first 1–2 weeks could therefore yield more physiologically representative data. However, measurements taken after days 7–14 should be viewed as a pragmatic stabilization point rather than a return to full physiological homeostasis.

While the onset of postpartum period is clearly defined, its end point remains less certain [105]. Although most physiologically systems return to their pre-pregnancy state within 6–12 months [106], many studies included (36 out of 61) concluded data collection ≤ 9 months postpartum. For example, studies reporting upward FMR trajectories among women with a preconception BMI > 25 kg/m² had follow-up limited to ≤ 2 months postpartum [2, 59], restricting conclusions on longer-term trajectories (Fig. 2c1). Longer follow up (≥ 12 months) may therefore be necessary since a substantial proportion of mothers retain the weight gained, even at one year postpartum [107, 108]. Additionally, this postpartum period may also overlap with the interpregnancy interval, i.e., the time between the delivery of the first pregnancy to the conception of the next pregnancy [109], thereby providing an appropriate endpoint for monitoring.

Measurement frequency should align with the study objectives, as association between a maternal factors and body composition are time dependent. For example, the relationship between breastfeeding and FM/FMR shifts overtime, with higher FM/FMR in exclusively breastfeeding women in early postpartum [55, 58, 59, 87], followed by variable trends thereafter [55, 58, 87]. These patterns may be explained by elevated prolactin and persistent insulin resistance [110–112] which together promote energy conservation and fat storage despite increased energy expenditure. Elevated prolactin during lactation promotes appetite, fat storage, and energy conservation, while reducing lipolysis and lowering estrogen levels, which may further slow metabolism [115]. Concurrently, persistent insulin resistance in some women—particularly following gestational diabetes or excess weight gain—results in elevated insulin levels that favour fat storage and inhibit lipid oxidation [112, 116]. Together, these mechanisms create a metabolic environment that supports fat retention, helping to explain postpartum weight retention despite increased energy demands [112–114].

Physical activity often increases with return to work [113, 114], potentially influencing the relationship between body composition measures and maternal factors. However, the impact of employment on body composition remains unclear due to lack of reporting on maternity leave and socioeconomic factors [115].

Finally, variations in FMR have been reported across ethnic groups [29, 30] (Fig. 6), potentially reflecting differences in diets, genetics, culture and social determinants [116, 117]. Parity and interpregnancy interval may further influence body composition through cumulative weight gain across pregnancies [118–120] and changes in activity patterns, as women often become more physically active following subsequent deliveries [121].

### Technique of measurement

Across the 86 included studies, 73 valid and reliable techniques were used to assess whole-body or regional body composition and/or anthropometry ranging from simple low-cost, non-invasive, rapid measurements (e.g. waist circumference, skinfold thickness at various sites, and BIA) [122, 123] to invasive, expensive, and more onerous techniques (e.g. magnetic resonance imaging, and DXA) [124, 125]. These also varied in burden, from time-intensive but low-cost approach (e.g. deuterium dilution) [126, 127], to rapid yet techniques (e.g., air displacement plethysmography) [128].

Fluctuations in fluid status during the early postpartum period can influence body composition measurements, particularly when using hydration-sensitive techniques such as BIA and DD [129, 130] influencing the estimation of FM and FMR. However, the trajectory patterns for FMR were consistent across measurement techniques, with similar proportion of studies reporting initial rapid reduction ( 71.4% overall and 75% for BIA ) as was the initial increase followed by a reduction (42.9% overall vs. 41.7% BIA). This consistency suggests that further stratification by assessment method was not warranted.

Nevertheless, early postpartum FMR/FM trends may still reflect technique-specific assumptions, although detailed methodological appraisal is beyond the scope of this review. Further, despite BIA’s sensitivity to fluid shifts, recent evidence indicates strong agreement with DXA, with only small, clinically acceptable differences – typically slight underestimation of FM and overestimate the FFM changes [129, 130].

In the postpartum period, selecting appropriate measurement techniques requires balancing the setting constraints (clinic versus field), staff training needs, and participant burden, including maternal time and procedural invasiveness. Consequently, rapid, low-cost methods such as skinfold thickness and circumference measurements are more advantageous. However, the measurement site must also be clinically relevant. For example, studies report rapid decline in central adiposity measured as waist circumference [23, 37, 54, 59, 63, 69] (Supplementary Fig. 3) and an increase in peripheral adiposity in triceps skinfold thickness [20, 22, 24, 44, 65, 66, 68, 80, 87, 88] (Fig. 4) in early postpartum. Although triceps skinfold thickness is commonly used, this may not be a preferential site as the increased FM may potentially be due to low levels of estrogen in the postpartum period that reduce the gynoid distribution of adipose tissue, promoting upper body and peripheral fat accumulation [7]. Therefore, selecting appropriate regional site is critical with waist circumference - a measure central adiposity- potentially representing a more clinically meaningful site [131, 132].

Additionally, central adiposity, as measured by waist circumference is strongly associated with CVD risk than BMI [133–135] and is also an independent predictor type 2 diabetes particularly in women [136]. Accordingly, the international guidelines recommend routine inclusion of waist circumference measurements alongside BMI to improve cardiometabolic risk assessment [137]. Emerging indices such as A Body Shape Index and Body Roundness Index, which integrate weight, height and waist circumference [138, 139] may further enhance cardiometabolic risk stratification beyond conventional measures [140]. Despite these advantages, the use of comprehensive body composition and anthropometric indices in clinical practice remains limited.

It is generally expected that weight and hence, by proxy, the body composition returns to the pre-pregnancy stage within the first postpartum year. However, only 10 studies reported pre-pregnancy body composition, and most did not specify the timing of measurement limiting the ability to determine when preconception status was reattained.

## Conclusion

This systematic review synthesised evidence on postpartum changes in body composition and anthropometric indices, and their associated factors, to identify the research gaps, and inform strategies to optimise maternal health. Although definitive conclusions remain limited, the review underscores the complexity and variability in postpartum body composition trajectories, influenced measurement timing, frequency, and duration of follow-up, maternal factors, and the body composition techniques used. Nevertheless, integrating body composition and specific anthropometric assessments alongside BMI measures may improve the understanding and management of postpartum adiposity.

## Supplementary Information

Supplementary Material 1.

Supplementary Material 2.

Supplementary Material 3.

Supplementary Material 4.

Supplementary Material 5.

Supplementary Material 6.

## Data Availability

All datasets including the search strategy, list of the included and excluded studies, data extracted, and quality assessment, are available in the Article, and its supplementary information, sheets and files.

## References

[1] Finlayson K, Crossland N, Bonet M, Downe S. What matters to women in the postnatal period: A meta-synthesis of qualitative studies. PLoS ONE. 2020;15(4):e0231415. 10.1371/journal.pone.0231415. 32320424 10.1371/journal.pone.0231415 PMC7176084

[2] Cho GJ, Yoon HJ, Kim EJ, Oh MJ, Seo HS, Kim HJ. Postpartum Changes in Body Composition. Obesity. 2011;19(12):2425–8. 10.1038/oby.2011.163. 21701569 10.1038/oby.2011.163

[3] De Lorenzo A, Gratteri S, Gualtieri P, Cammarano A, Bertucci P, Di Renzo L. Why primary obesity is a disease? J Transl Med. 2019;17(1):169. 10.1186/s12967-019-1919-y. 31118060 10.1186/s12967-019-1919-y PMC6530037

[4] Gridneva Z, Rea A, Hepworth AR, Ward LC, Lai CT, Hartmann PE, et al. Relationships between Breastfeeding Patterns and Maternal and Infant Body Composition over the First 12 Months of Lactation. Nutrients. 2018;10(1). 10.3390/nu10010045. 10.3390/nu10010045 PMC5793273 29303992

[5] Malpeli A, Mansur JL, De Santiago S, Villalobos R, Armanini A, Apezteguía M, et al. Changes in bone mineral density of adolescent mothers during the 12-month postpartum period. Public Health Nutr. 2010;13(10):1522–7. 10.1017/S1368980009992199. 19954573 10.1017/S1368980009992199

[6] Flor-Alemany M, Acosta-Manzano P, Migueles JH, Henriksson P, Löf M, Aparicio VA. Impact of Exercise Intervention Combined with Optimal Mediterranean Diet Adherence during Pregnancy on Postpartum Body Composition: A Quasi-Experimental Study—The GESTAFIT Project. Nutrients. 2023;15(20):4413. 37892487 10.3390/nu15204413 PMC10609918

[7] Butte NF, Hopkinson JM. Body Composition Changes during Lactation Are Highly Variable among Women1,2. J Nutr. 1998;128(2):S381–5. 10.1093/jn/128.2.381S. 10.1093/jn/128.2.381S 9478031

[8] Suntio K, Saarelainen H, Laitinen T, Valtonen P, Heiskanen N, Lyyra-Laitinen T, et al. Women with hypertensive pregnancies have difficulties in regaining pre-pregnancy weight and show metabolic disturbances. Obes (Silver Spring). 2010;18(2):282–6. 10.1038/oby.2009.262. 10.1038/oby.2009.262 19696762

[9] National Research Council. Weight Gain During Pregnancy: Reexamining the Guidelines. Washington DC; 2009. 20669500

[10] He X, Zhu M, Hu C, Tao X, Li Y, Wang Q, et al. Breast-feeding and postpartum weight retention: a systematic review and meta-analysis. Public Health Nutr. 2015;18(18):3308–16. 10.1017/S1368980015000828. 25895506 10.1017/S1368980015000828 PMC10271764

[11] Ruchat S-M, Mottola MF, Skow RJ, Nagpal TS, Meah VL, James M, et al. Effectiveness of exercise interventions in the prevention of excessive gestational weight gain and postpartum weight retention: a systematic review and meta-analysis. Br J Sports Med. 2018;52(21):1347–56. 10.1136/bjsports-2018-099399. 30337461 10.1136/bjsports-2018-099399

[12] van der Pligt P, Willcox J, Hesketh KD, Ball K, Wilkinson S, Crawford D, et al. Systematic review of lifestyle interventions to limit postpartum weight retention: implications for future opportunities to prevent maternal overweight and obesity following childbirth. Obes Rev. 2013;14(10):792–805. 10.1111/obr.12053. 23773448 10.1111/obr.12053

[13] Etchison WC, Bloodgood EA, Minton CP, Thompson NJ, Collins MA, Hunter SC, et al. Body mass index and percentage of body fat as indicators for obesity in an adolescent athletic population. Sports Health. 2011;3(3):249–52. 10.1177/1941738111404655. 23016014 10.1177/1941738111404655 PMC3445161

[14] Biddulph C, Holmes M, Kuballa A, Carter RJ, Maher J. Beyond the BMI: Validity and Practicality of Postpartum Body Composition Assessment Methods during Lactation: A Scoping Review. Nutrients. 2022;14(11):2197. 35683995 10.3390/nu14112197 PMC9182963

[15] World Health Organisation. Recommendations Geneva: World Health Organization. 2024 [Available from: https://www.who.int/health-topics/breastfeeding#tab=tab_2

[16] Amorim Adegboye AR, Linne YM. Diet or exercise, or both, for weight reduction in women after childbirth. Cochrane Database Syst Rev. 2013;2013(7):Cd005627. 10.1002/14651858.CD005627.pub3. 23881656 10.1002/14651858.CD005627.pub3 PMC9392837

[17] Amorim AR, Linne YM, Lourenco PM. Diet or exercise, or both, for weight reduction in women after childbirth. Cochrane Database Syst Rev. 2007;3Cd005627. 10.1002/14651858.CD005627.pub2. 10.1002/14651858.CD005627.pub2 17636810

[18] Meera Viswanathan M, Berkman DN, Dryden DM, Hartling L. Assessing Risk of Bias and Confounding in Observational Studies of Interventions or Exposures: Further Development of the RTI Item Bank. Rockville: Agency for Healthcare Research and Quality (US); 2013. 24006553

[19] Barry VG, Martin SL, Chandler-Laney P, Carter EB, Worthington CS. A Comparison of Bioimpedance Analysis vs. Dual X-ray Absorptiometry for Body Composition Assessment in Postpartum Women and Non-Postpartum Controls. Int J Environ Res Public Health. 2022;19(20):10. 10.3390/ijerph192013636. 10.3390/ijerph192013636 PMC9602548 36294216

[20] Papathakis PC, Van Loan MD, Rollins NC, Chantry CJ, Bennish ML, Brown KH. Body composition changes during lactation in HIV-infected and HIV-uninfected South African women. J Acquir Immune Defic Syndr. 2006;43(4):467–74. 10.1097/01.qai.0000243094.42276.92. 16980904 10.1097/01.qai.0000243094.42276.92

[21] Versele V, Stas L, Aerenhouts D, Deliens T, Clarys P, Gucciardo L, et al. Changes in maternal and paternal body composition during the transition to parenthood (TRANSPARENTS). Obes (Silver Spring). 2023;31(1):225–33. 10.1002/oby.23586. 10.1002/oby.23586 36471905

[22] Widen EM, Tsai I, Collins SM, Wekesa P, China J, Krumdieck N, et al. HIV infection and increased food insecurity are associated with adverse body composition changes among pregnant and lactating Kenyan women. Eur J Clin Nutr. 2019;73(3):474–82. 10.1038/s41430-018-0285-9. 30185898 10.1038/s41430-018-0285-9 PMC6668710

[23] Kajale NA, Khadilkar VV, Mughal Z, Chiplonkar SA, Khadilkar AV. Changes in body composition of Indian lactating women: a longitudinal study. Asia Pac J Clin Nutr. 2016;25(3):556–62. 10.6133/apjcn.092015.16. 27440691 10.6133/apjcn.092015.16

[24] Guzmán-Mercado E, Vásquez-Garibay EM, Sánchez Ramírez CA, Muñoz-Esparza NC, Larrosa-Haro A, Meza Arreola PL. Full Breastfeeding Modifies Anthropometric and Body Composition Indicators in Nursing Mothers. Breastfeed Med. 2021;16(3):264–71. 10.1089/bfm.2020.0144. 33179962 10.1089/bfm.2020.0144

[25] Kramer FM, Stunkard AJ, Marshall KA, McKinney S, Liebschutz J. Breast-feeding reduces maternal lower-body fat. J Am Diet Assoc. 1993;93(4):429–33. 10.1016/0002-8223(93)92289-a. 8454811 10.1016/0002-8223(93)92289-a

[26] Ryan JT, Day H, Egger MJ, Wu J, Depner CM, Shaw JM. Night-time sleep duration and postpartum weight retention in primiparous women. Sleep Adv. 2024;5(1):zpad056. 10.1093/sleepadvances/zpad056. 38314118 10.1093/sleepadvances/zpad056 PMC10838128

[27] AbuSabha R, Greene G. Body weight, body composition, and energy intake changes in breastfeeding mothers. J Hum Lact. 1998;14(2):119–24. 10.1177/089033449801400211. 9775844 10.1177/089033449801400211

[28] Piers LS, Diggavi SN, Thangam S, van Raaij JM, Shetty PS, Hautvast JG. Changes in energy expenditure, anthropometry, and energy intake during the course of pregnancy and lactation in well-nourished Indian women. Am J Clin Nutr. 1995;61(3):501–13. 10.1093/ajcn/61.3.501. 7872213 10.1093/ajcn/61.3.501

[29] Tohill BC, McCrory MA, Nommsen-Rivers LA, Dewey KG. Postpartum changes in body composition: Differences associated with ethnicity and breastfeeding. Faseb J. 2000;14(4):A531–A.

[30] Wang X, Kishman EE, Liu J, Castleberry LA, McLain A, Sparks JR, et al. Body weight and fat trajectories of Black and White women in the first postpartum year. Obesity. 2023;31(6):1655–65. 10.1002/oby.23724. 37169733 10.1002/oby.23724 PMC10198894

[31] Butte NF, Hopkinson JM, Mehta N, Moon JK, Smith EO. Adjustments in energy expenditure and substrate utilization during late pregnancy and lactation. Am J Clin Nutr. 1999;69(2):299–307. 10.1093/ajcn/69.2.299. 9989696 10.1093/ajcn/69.2.299

[32] Nyirati CM, Habash DL, Shaffer LE. Weight and body fat changes in postpartum depot-medroxyprogesterone acetate users. Contraception. 2013;88(1):169–76. 10.1016/j.contraception.2012.10.016. 23177262 10.1016/j.contraception.2012.10.016

[33] Moller UK, Streym SV, Mosekilde L, Rejnmark L. Changes in bone mineral density and body composition during pregnancy and postpartum. A controlled cohort study. Osteoporos Int. 2012;23(4):1213–23. 10.1007/s00198-011-1654-6. 21607805 10.1007/s00198-011-1654-6

[34] Kyle EM, Larson-Meyer E. Effect of physical activity and dietary intake on bone mineral density in lactating postpartum women. 2014(1566341):83. doi.

[35] Kane HS, Brown JA, Nelson JA, Cha L, Schetter CD, Robles TF. Social relationships and cardiometabolic risk in low-income mothers following birth. Health Psychol. 2025;44(5):426–35. 10.1037/hea0001422. 40232778 10.1037/hea0001422 PMC12400509

[36] Phaloprakarn C, Suthasmalee S, Tangjitgamol S. Impact of postpartum weight change on metabolic syndrome and its components among women with recent gestational diabetes mellitus. Reproductive Health. 2024;21(1):1–10. 10.1186/s12978-024-01783-4. 38582891 10.1186/s12978-024-01783-4 PMC10998404

[37] Souza MDDC, Ribeiro MM, Ferreira LB, do Carmo AS, dos Santos LC. Weight Reduction and Changes in Body Circumferences in Lactating Women as a Function of Differences in Dietary Macronutrient Content. Breastfeed Med. 2022;17(6):511–8. 10.1089/bfm.2021.0354. 35353584 10.1089/bfm.2021.0354

[38] Dugdale AE, Eaton-Evans J. The effect of lactation and other factors on post-partum changes in body-weight and triceps skinfold thickness. Br J Nutr. 1989;61(2):149–53. 10.1079/bjn19890105. 2706221 10.1079/bjn19890105

[39] Sidebottom AC, Brown JE, Jacobs DR. Jr. Pregnancy-related changes in body fat. Eur J Obstet Gynecol Reprod Biol. 2001;94(2):216–23. 10.1016/s0301-2115(00)00329-8. 11165728 10.1016/s0301-2115(00)00329-8

[40] Fornes NS, Dorea JG. Subcutaneous fat changes in low-income lactating mothers and growth of breast-fed infants. J Am Coll Nutr. 1995;14(1):61–5. 10.1080/07315724.1995.10718474. 7706612 10.1080/07315724.1995.10718474

[41] Halland Nesse S, Ottestad I, Winkvist A, Bertz F, Ellegård L, Brekke HK. Predictive equations for estimating resting energy expenditure in women with overweight and obesity at three postpartum stages. J Nutr Sci. 2020;9:e31. 10.1017/jns.2020.16. 32913643 10.1017/jns.2020.16 PMC7443793

[42] Howard K, Maples JM, Tinius RA. Modifiable Maternal Factors and Their Relationship to Postpartum Depression. Int J Environ Res Public Health. 2022;19(19). 10.3390/ijerph191912393. 10.3390/ijerph191912393 PMC9564437 36231692

[43] Kinkade CW, Rivera-Núñez Z, Thurston SW, Kannan K, Miller RK, Brunner J, et al. Per- and polyfluoroalkyl substances, gestational weight gain, postpartum weight retention and body composition in the UPSIDE cohort. Environ Health Global Access Sci Sour. 2023;22(1). 10.1186/s12940-023-01009-3. 10.1186/s12940-023-01009-3 PMC10474772 37658449

[44] Rashid M, Ulijaszek SJ. Daily energy expenditure across the course of lactation among urban Bangladeshi women. Am J Phys Anthropol. 1999;110(4):457–65. 10.1002/(sici)1096-8644(199912)110:4<457::Aid-ajpa6>3.0.Co;2-x. 10564575 10.1002/(SICI)1096-8644(199912)110:4<457::AID-AJPA6>3.0.CO;2-X

[45] Schneider C, Chandler-Laney PC. Postpartum Body Composition Breast Milk Horm. 2019(13899262):126. doi.

[46] Sohlström A, Forsum E. Changes in total body fat during the human reproductive cycle as assessed by magnetic resonance imaging, body water dilution, and skinfold thickness: a comparison of methods. Am J Clin Nutr. 1997;66(6):1315–22. 10.1093/ajcn/66.6.1315. 9394681 10.1093/ajcn/66.6.1315

[47] Sohlström A, Wahlund LO, Forsum E. Total body fat and its distribution during human reproduction as assessed by magnetic resonance imaging. Basic Life Sci. 1993;60:181–4. 10.1007/978-1-4899-1268-8_41. 8110105 10.1007/978-1-4899-1268-8_41

[48] Brewer MM, Wozniak P, Bates MR, Vannoy LP, Mangum M. Assessment of postpartum body fat change from underwater weight and skinfold thickness measurements. Nutr Res. 1989;9(12):1331–7. 10.1016/S0271-5317(89)80157-5.

[49] Bzikowska A, Czerwonogrodzka-Senczyna A, Weker H, Wesołowska A. Correlation between human milk composition and maternal nutritional status. Rocz Panstw Zakl Hig. 2018;69(4):363–7. 10.32394/rpzh.2018.0041. 30525326 10.32394/rpzh.2018.0041

[50] van Raaij JM, Schonk CM, Vermaat-Miedema SH, Peek ME, Hautvast JG. Body fat mass and basal metabolic rate in Dutch women before, during, and after pregnancy: a reappraisal of energy cost of pregnancy. Am J Clin Nutr. 1989;49(5):765–72. 10.1093/ajcn/49.5.765. 2718913 10.1093/ajcn/49.5.765

[51] Cortés-Martín A, Romo-Vaquero M, García-Mantrana I, Rodríguez-Varela A, Collado MC, Espín JC, et al. Urolithin metabotypes can anticipate the different restoration of the gut microbiota and anthropometric profiles during the first year postpartum. Nutrients. 2019;11(9). 10.3390/nu11092079. 10.3390/nu11092079 PMC6769946 31484413

[52] Widen EM, Collins SM, Khan H, Biribawa C, Acidri D, Achoko W, et al. Food insecurity, but not HIV-infection status, is associated with adverse changes in body composition during lactation in Ugandan women of mixed HIV status. Am J Clin Nutr. 2017;105(2):361–8. 10.3945/ajcn.116.142513. 28052888 10.3945/ajcn.116.142513 PMC5267304

[53] Nartea R, Mitoiu BI, Nica AS. Correlation between Pregnancy Related Weight Gain, Postpartum Weight loss and Obesity: a Prospective Study. J Med Life. 2019;2019(2):178–83. 10.25122/jml-2019-0015. 10.25122/jml-2019-0015 PMC6685304 31406521

[54] Kajale N, Khadilkar V, Chiplonkar S, Padidela R, Khadilkar A. Prevalence of metabolic syndrome markers among women at 1-year postpartum as per prepregnancy body mass index status: A longitudinal study. Indian J Endocrinol Metab. 2017;21(5):703–9. 10.4103/ijem.IJEM_145_17. 28989878 10.4103/ijem.IJEM_145_17 PMC5628540

[55] Rabi B, Benjeddou K, Idrissi M, Rami A, Mekkaoui B, El Hamdouchi A, et al. Effects of Breastfeeding on Maternal Body Composition in Moroccan Lactating Women during Twelve Months after Birth Using Stable Isotopic Dilution Technique. Nutrients. 2021;13(1). 10.3390/nu13010146. 10.3390/nu13010146 PMC7823570 33406595

[56] Ramiro-Cortijo D, Singh P, Herranz Carrillo G, Gila-Díaz A, Martín-Cabrejas MA, Martin CR, et al. Association of maternal body composition and diet on breast milk hormones and neonatal growth during the first month of lactation. Front Endocrinol (Lausanne). 2023;14:1090499. 10.3389/fendo.2023.1090499. 36936154 10.3389/fendo.2023.1090499 PMC10018215

[57] Domer MC, Beerman KA, Ahmadzadeh A, Dasgupta N, Williams JE, McGuire MA, et al. Loss of body fat and associated decrease in leptin in early lactation are related to shorter duration of postpartum anovulation in healthy US women. J Hum Lact. 2015;31(2):282–93. 10.1177/0890334414565794. 25596410 10.1177/0890334414565794

[58] Brewer MM, Bates MR, Vannoy LP. Postpartum changes in maternal weight and body fat depots in lactating vs nonlactating women. Am J Clin Nutr. 1989;49(2):259–65. 10.1093/ajcn/49.2.259. 2916446 10.1093/ajcn/49.2.259

[59] Aydin B, Yalçin SS. Changes in maternal anthropometric measurements in the first postpartum month and associated factors. Am J Hum Biol. 2022;34(1). 10.1002/ajhb.23580. 10.1002/ajhb.23580 33598996

[60] Mayo N, Fernandez D. Measurement and Evaluation of Seated Behaviors and Markers of Adiposity in a Postpartum Cohort. 2022(29068231):96. doi.

[61] Kac G, Benicio M, Velasquez-Melendez G, Valente JG, Struchiner CJ. Gestational weight gain and prepregnancy weight influence postpartum weight retention in a cohort of Brazilian women. J Nutr. 2004;134(3):661–6. 10.1093/jn/134.3.661. 14988464 10.1093/jn/134.3.661

[62] Kosinski C, Rossel JB, Gross J, Helbling C, Quansah DY, Collet TH, et al. Adverse metabolic outcomes in the early and late postpartum after gestational diabetes are broader than glucose control. BMJ Open Diabetes Res Care. 2021;9(2). 10.1136/bmjdrc-2021-002382. 10.1136/bmjdrc-2021-002382 PMC8576469 34750153

[63] Ahmadibeni A, Kashani P, Hallaj MS, Ghanbari S, Javadifar N. The relationship of pre-pregnancy body mass index with maternal anthropometric indices, weight retention and the baby’s weight and nutrition in the first 6 months post-partum. BMC Pregnancy Childbirth. 2023;23(1):802. 10.1186/s12884-023-06116-0. 37986057 10.1186/s12884-023-06116-0 PMC10662692

[64] Motoyasu NL, Berger PK, Anderson AK, Smith JJ, Birch LL. Infant feeding mode is associated with differences in postpartum weight retention and percent body fat from 2 to 16 weeks. Faseb J. 2017;31(1 Supplement 1). doi.

[65] Barbosa L, Butte NF, Villalpando S, Wong WW, Smith EO. Maternal energy balance and lactation performance of Mesoamerindians as a function of body mass index. Am J Clin Nutr. 1997;66(3):575–83. 10.1093/ajcn/66.3.575. 9280176 10.1093/ajcn/66.3.575

[66] Martínez H, Allen LH, Lung’aho M, Chávez A, Pelto GH. Maternal fatness in Mexican women predicts body composition changes in pregnancy and lactation. Adv Exp Med Biol. 1994;352:99–107. 10.1007/978-1-4899-2575-6_7. 7832062 10.1007/978-1-4899-2575-6_7

[67] Butte NF, Ellis KJ, Wong WW, Hopkinson JM, Smith EO. Composition of gestational weight gain impacts maternal fat retention and infant birth weight. Am J Obstet Gynecol. 2003;189(5):1423–32. 10.1067/s0002-9378(03)00596-9. 14634581 10.1067/s0002-9378(03)00596-9

[68] Manning-Dalton C, Allen LH. The effects of lactation on energy and protein consumption, postpartum weight change and body compostion of well nourished North American women. Nutr Res. 1983;3(3):293–308. 10.1016/S0271-5317(83)80077-3.

[69] Chou TW, Chan GM, Moyer-Mileur L. Postpartum body composition changes in lactating and non-lactating primiparas. Nutrition. 1999;15(6):481–4. 10.1016/s0899-9007(99)00055-6. 10378204 10.1016/s0899-9007(99)00055-6

[70] Villalpando S, Flores-Huerta S, Fajardo A, Hernandez-Beltran MJ. Ethanol consumption during pregnancy and lactation. Changes in the nutritional status of predominantly breastfeeding mothers. Arch Med Res. 1993;24(4):333–8. 8118156

[71] Butte NF, Hopkinson JM, Ellis KJ, Wong WW, Smith EO. Changes in fat-free mass and fat mass in postpartum women: a comparison of body composition models. Int J Obes Relat Metab Disord. 1997;21(10):874–80. 10.1038/sj.ijo.0800482. 9347405 10.1038/sj.ijo.0800482

[72] Izumi M, Manabe E, Uematsu S, Watanabe A, Moritani T. Changes in autonomic nervous system activity, body weight, and percentage fat mass in the first year postpartum and factors regulating the return to pre-pregnancy weight. J Physiol Anthropol. 2016;35(1):26. 10.1186/s40101-016-0115-5. 27788690 10.1186/s40101-016-0115-5 PMC5081745

[73] Guindre N. Maternal characteristics related to post partum body composition changes among lactating and non-lactating mothers [M.Sc]. Scotland: University of Glasgow (United Kingdom); 1989.

[74] Osoti AO, Page ST, Richardson BA, Guthrie BL, Kinuthia J, Polyak SJ, et al. Postpartum metabolic syndrome and high-sensitivity C-reactive protein after gestational hypertension and pre-eclampsia. Int J Gynecol Obstet. 2020;151(3):443–9. 10.1002/ijgo.13352. 10.1002/ijgo.13352 PMC7722223 32812650

[75] Hendrycks R, Yang M, Hitchcock R, Leitner M, Niederauer S, Nygaard IE, et al. Temporal trends in trunk flexor endurance and intra-abdominal pressure in postpartum women. Physiother Theory Pract. 2021;37(11):1217–26. 10.1080/09593985.2019.1686792. 31686567 10.1080/09593985.2019.1686792 PMC7198326

[76] Retnakaran R, Qi Y, Sermer M, Connelly PW, Hanley AJG, Zinman B. β-cell function declines within the first year postpartum in women with recent glucose intolerance in pregnancy. Diabetes Care. 2010;33(8):1798–804. 10.2337/dc10-0351. 20484133 10.2337/dc10-0351 PMC2909065

[77] Osoti AO, Page ST, Richardson BA, Guthrie BL, Kinuthia J, Polyak SJ, et al. Postpartum metabolic syndrome after gestational hypertension and preeclampsia, a prospective cohort study. Pregnancy Hypertens. 2019;18:35–41. 10.1016/j.preghy.2019.08.088. 31493627 10.1016/j.preghy.2019.08.088 PMC6884686

[78] Hajimirzaie SS, Tehranian N, Golabpour A, Khosravi A, Mousavi SA, Keramat A, et al. Predicting postpartum female sexual interest/arousal disorder via adiponectin and biopsychosocial factors: a cohort-based decision tree study. Sci Rep. 2025;15(1):27354. 10.1038/s41598-025-12025-3. 40717115 10.1038/s41598-025-12025-3 PMC12301432

[79] Kulkarni B, Shatrugna V, Nagalla B, Ajeya Kumar P, Usha Rani K, Chandrakala Omkar A. Maternal weight and lean body mass may influence the lactation-related bone changes in young undernourished Indian women. Br J Nutr. 2009;101(10):1527–33. 10.1017/s0007114508084067. 18959808 10.1017/S0007114508084067

[80] Piperata BA, Dufour DL. Diet, energy expenditure, and body composition of lactating Ribeirinha women in the Brazilian Amazon. Am J Hum Biol. 2007;19(5):722–34. 10.1002/ajhb.20628. 17657725 10.1002/ajhb.20628

[81] Vinoy S, Rosetta L, Mascie-Taylor CG. Repeated measurements of energy intake, energy expenditure and energy balance in lactating Bangladeshi mothers. Eur J Clin Nutr. 2000;54(7):579–85. 10.1038/sj.ejcn.1601060. 10918469 10.1038/sj.ejcn.1601060

[82] Berggren EK, Presley L, Amini SB, Hauguel-de Mouzon S, Catalano PM. Are the metabolic changes of pregnancy reversible in the first year postpartum? Diabetologia. 2015;58(7):1561–8. 10.1007/s00125-015-3604-x. 25957777 10.1007/s00125-015-3604-x PMC4703315

[83] González HF, Malpeli A, Mansur JL, De Santiago S, Etchegoyen GS. Changes in body composition in lactating adolescent mothers. Arch Latinoam Nutr. 2005;55(3):252–6. 16454051

[84] Elliott SA, Pereira LCR, McCargar LJ, Prado CM, Bell RC. Trajectory and determinants of change in lean soft tissue over the postpartum period. Br J Nutr. 2019;121(10):1137–45. 10.1017/s0007114518002015. 30178726 10.1017/S0007114518002015

[85] Pereira LCR, Elliott SA, McCargar LJ, Bell RC, Vu K, Bell G, et al. The influence of energy metabolism on postpartum weight retention. Am J Clin Nutr. 2019;109(6):1588–99. 10.1093/ajcn/nqy389. 31075789 10.1093/ajcn/nqy389

[86] Sadurskis A, Kabir N, Wager J, Forsum E. Energy metabolism, body composition, and milk production in healthy Swedish women during lactation. Am J Clin Nutr. 1988;48(1):44–9. 10.1093/ajcn/48.1.44. 3389329 10.1093/ajcn/48.1.44

[87] Motil KJ, Sheng HP, Kertz BL, Montandon CM, Ellis KJ. Lean body mass of well-nourished women is preserved during lactation. Am J Clin Nutr. 1998;67(2):292–300. 10.1093/ajcn/67.2.292. 9459378 10.1093/ajcn/67.2.292

[88] Butte NF, Garza C, Stuff JE, Smith EO, Nichols BL. Effect of maternal diet and body composition on lactational performance. Am J Clin Nutr. 1984;39(2):296–306. 10.1093/ajcn/39.2.296. 6695830 10.1093/ajcn/39.2.296

[89] Dufour DL, Reina JC, Spurr GB. Energy intake and expenditure of free-living, lactating Colombian women in an urban setting. Eur J Clin Nutr. 2002;56(3):205–13. 10.1038/sj.ejcn.1601302. 11960295 10.1038/sj.ejcn.1601302

[90] Opala-Berdzik A, Błaszczyk JW, Świder D, Cieślińska-Świder J. Trunk forward flexion mobility in reference to postural sway in women after delivery: A prospective longitudinal comparison between early pregnancy and 2- and 6-month postpartum follow-ups. Clin Biomech (Bristol Avon). 2018;56:70–4. 10.1016/j.clinbiomech.2018.05.009. 10.1016/j.clinbiomech.2018.05.009 29807274

[91] Caire G, Casanueva E, De Regil LM, Bolaños A, de la Calderón AM. Nutritional status of exclusively breastfeeding adolescents from northwest and central Mexico. Adv Exp Med Biol. 2004;554:337–9. 10.1007/978-1-4757-4242-8_35. 15384595 10.1007/978-1-4757-4242-8_35

[92] Caire-Juvera G, Casanueva E, Bolaños-Villar AV, de Regil LM, de la Calderón AM. No changes in weight and body fat in lactating adolescent and adult women from Mexico. Am J Hum Biol. 2012;24(4):425–31. 10.1002/ajhb.22234. 22344621 10.1002/ajhb.22234

[93] Forsum E, Sadurskis A, Wager J. Estimation of body fat in healthy Swedish women during pregnancy and lactation. Am J Clin Nutr. 1989;50(3):465–73. 10.1093/ajcn/50.3.465. 2773826 10.1093/ajcn/50.3.465

[94] Forsum E, Henriksson P, Löf M. The two-component model for calculating total body fat from body density: an evaluation in healthy women before, during and after pregnancy. Nutrients. 2014;6(12):5888–99. 10.3390/nu6125888. 25526240 10.3390/nu6125888 PMC4277005

[95] South-Paul JE, Rajagopal KR, Tenholder MF. Exercise responses prior to pregnancy and in the postpartum state. Med Sci Sports Exerc. 1992;24(4):410–4. 1560735

[96] Guillermo-Tuazon MA, Barba CV, van Raaij JM, Hautvast JG. Energy intake, energy expenditure, and body composition of poor rural Philippine women throughout the first 6 mo of lactation. Am J Clin Nutr. 1992;56(5):874–80. 10.1093/ajcn/56.5.874. 1415006 10.1093/ajcn/56.5.874

[97] Kopp-Hoolihan LE, van Loan MD, Wong WW, King JC. Longitudinal assessment of energy balance in well-nourished, pregnant women. Am J Clin Nutr. 1999;69(4):697–704. 10.1093/ajcn/69.4.697. 10197571 10.1093/ajcn/69.4.697

[98] Quandt SA. Changes in maternal postpartum adiposity and infant feeding patterns. Am J Phys Anthropol. 1983;60(4):455–61. 6846516 10.1002/ajpa.1330600407

[99] Department of Making Pregnancy Safer. WHO Technical Consultation on Postpartum and Postnatal Care. Geneva: World Health Organization; 2010. p. 57. 26269861

[100] Girandola RN, Wiswell RA, Romero G. Body Composition Changes Resulting from Fluid Ingestion and Dehydration. Research Quarterly American Alliance for Health. Phys Educ Recreation. 1977;48(2):299–303. 10.1080/10671315.1977.10615425. 267969

[101] Lukaski HC, Hall CB, Siders WA. Assessment of change in hydration in women during pregnancy and postpartum with bioelectrical impedance vectors. Nutrition. 2007;23(7–8):543–50. 10.1016/j.nut.2007.05.001. 17570642 10.1016/j.nut.2007.05.001

[102] Maternal Physiological Changes During Pregnancy. Labor, and the Postpartum Period. In: Datta S, editor. Obstetric Anesthesia Handbook. New York, NY: Springer New York; 2006. pp. 1–13.

[103] Cheung KL, Lafayette RA. Renal physiology of pregnancy. Adv Chronic Kidney Dis. 2013;20(3):209–14. 10.1053/j.ackd.2013.01.012. 23928384 10.1053/j.ackd.2013.01.012 PMC4089195

[104] Romm A. CHAPTER 17 - The Postpartum. In: Romm A, Hardy ML, Mills S, editors. Botanical Medicine for Women’s Health. Saint Louis: Churchill Livingstone; 2010. pp. 416–32.

[105] Kumarasinghe M, Herath MP, Hills AP, Ahuja KDK. Postpartum versus postnatal period: Do the name and duration matter? PLoS ONE. 2024;19(4):e0300118. 10.1371/journal.pone.0300118. 38669219 10.1371/journal.pone.0300118 PMC11051636

[106] Berens P. Overview of the postpartum period: Normal physiology and routine maternal care: Wolter Kluwer; 2023 [Available from: https://www.uptodate.com/contents/overview-of-the-postpartum-period-normal-physiology-and-routine-maternal-care#references

[107] Lipsky LM, Strawderman MS, Olson CM. Maternal weight change between 1 and 2 years postpartum: the importance of 1 year weight retention. Obes (Silver Spring). 2012;20(7):1496–502. 10.1038/oby.2012.41. 10.1038/oby.2012.41 PMC4862000 22334257

[108] Endres LK, Straub H, McKinney C, Plunkett B, Minkovitz CS, Schetter CD, et al. Postpartum weight retention risk factors and relationship to obesity at 1 year. Obstet Gynecol. 2015;125(1):144–52. 10.1097/aog.0000000000000565. 25560116 10.1097/AOG.0000000000000565 PMC4286308

[109] Dorney E, Mazza D, Black KI. Interconception care. Australian J Gen Pract. 2020;49(6):317–22. 32464729 10.31128/AJGP-02-20-5242

[110] Karcz K, Gaweł P, Królak-Olejnik B. Evaluation of Maternal Factors Affecting Postpartum Insulin Resistance Markers in Mothers with Gestational Diabetes-A Case-Control Study. Nutrients. 2024;16(22). 10.3390/nu16223871. 10.3390/nu16223871 PMC11597180 39599657

[111] Lopez-Vicchi F, De Winne C, Ornstein AM, Sorianello E, Toneatto J, Becu-Villalobos D. Severe Hyperprolactinemia Promotes Brown Adipose Tissue Whitening and Aggravates High Fat Diet Induced Metabolic Imbalance. Front Endocrinol (Lausanne). 2022;13:883092. 10.3389/fendo.2022.883092. 35757410 10.3389/fendo.2022.883092 PMC9226672

[112] Nilsson L, Binart N, Bohlooly YM, Bramnert M, Egecioglu E, Kindblom J, et al. Prolactin and growth hormone regulate adiponectin secretion and receptor expression in adipose tissue. Biochem Biophys Res Commun. 2005;331(4):1120–6. 10.1016/j.bbrc.2005.04.026. 15882993 10.1016/j.bbrc.2005.04.026

[113] Stuebe AM, Rich-Edwards JW. The reset hypothesis: lactation and maternal metabolism. Am J Perinatol. 2009;26(1):81–8. 10.1055/s-0028-1103034. 19031350 10.1055/s-0028-1103034 PMC3006166

[114] Guardino CM, Hobel CJ, Shalowitz MU, Ramey SL, Dunkel Schetter C. Community Child Health N. Psychosocial and demographic predictors of postpartum physical activity. J Behav Med. 2018;41(5):668–79. 10.1007/s10865-018-9931-x. 29740746 10.1007/s10865-018-9931-x PMC6814308

[115] Franzoi IG, Sauta MD, De Luca A, Granieri A. Returning to work after maternity leave: a systematic literature review. Arch Womens Ment Health. 2024;27(5):737–49. 10.1007/s00737-024-01464-y. 38575816 10.1007/s00737-024-01464-y PMC11405436

[116] Heymsfield SB, Peterson CM, Thomas DM, Heo M, Schuna JM. Jr. Why are there race/ethnic differences in adult body mass index-adiposity relationships? A quantitative critical review. Obes Rev. 2016;17(3):262–75. 10.1111/obr.12358. 26663309 10.1111/obr.12358 PMC4968570

[117] Rogol AD. Growth, body composition and hormonal axes in children and adolescents. J Endocrinol Invest. 2003;26(9):855–60. 10.1007/bf03345236. 14964438 10.1007/BF03345236

[118] Hill B, Bergmeier H, McPhie S, Fuller-Tyszkiewicz M, Teede H, Forster D, et al. Is parity a risk factor for excessive weight gain during pregnancy and postpartum weight retention? A systematic review and meta‐analysis. Obes Rev. 2017;18(7):755–64. 10.1111/obr.12538. 28512991 10.1111/obr.12538

[119] Harris HE, Ellison GTH, Holliday M. Is there an independent association between parity and maternal weight gain? Ann Hum Biol. 1997;24(6):507–19. 10.1080/03014469700005272. 9395736 10.1080/03014469700005272

[120] Davis EM, Babineau DC, Wang X, Zyzanski S, Abrams B, Bodnar LM, et al. Short Inter-pregnancy Intervals, Parity, Excessive Pregnancy Weight Gain and Risk of Maternal Obesity. Matern Child Health J. 2014;18(3):554–62. 10.1007/s10995-013-1272-3. 23595566 10.1007/s10995-013-1272-3 PMC3840151

[121] Nomura Y, Araki T. Factors influencing physical activity in postpartum women during the COVID-19 pandemic: a cross-sectional survey in Japan. BMC Womens Health. 2022;22(1):371. 10.1186/s12905-022-01959-9. 36076222 10.1186/s12905-022-01959-9 PMC9454407

[122] Tzotzas T, Krassas GE, Doumas A. [Body composition analysis in obesity: radionuclide and non radionuclide methods]. Hell J Nucl Med. 2008;11(1):63–71. 18392235

[123] Michener J, Lam S, Kolesnik S, Thornton JC, Wang J, Pierson JR. RN. Skinfolds versus Bioimpedance Analysis for Predicting Fat-free Mass. Ann N Y Acad Sci. 2000;904(1):339–41. 10.1111/j.1749-6632.2000.tb06478.x. 10865767 10.1111/j.1749-6632.2000.tb06478.x

[124] Karaba-Jakovljevic D. Assessment methods of body composition. Praxis Med. 2016;45(3–4):71–7. 10.5937/pramed1604071k.

[125] Pietrobelli A, Brambilla P. Body composition measurements. Ital J Pediatr. 2014;40(1):A6. 10.1186/1824-7288-40-S1-A6.

[126] Chapman CE, Wilkinson PS, Murphy MR, Erickson PS. Technical note: Evaluating nuclear magnetic resonance spectroscopy for determining body composition in Holstein dairy calves using deuterium oxide dilution methods. J Dairy Sci. 2017;100(4):2807–11. 10.3168/jds.2016-11888. 28161168 10.3168/jds.2016-11888

[127] Hooper SE, Eshelman AN, Cowan AN, Roistacher A, Paneitz TS, Amelon SK. Using Deuterium Oxide as a Non-Invasive, Non-Lethal Tool for Assessing Body Composition and Water Consumption in Mammals: 1940-087X; 2020. e59442. 10.3791/59442 PMC10228182 32150166

[128] Shaw G, Kerr A. Non-imaging Method: Air Displacement Plethysmography (Bod Pod). In: Hume PA, Kerr DA, Ackland TR, editors. Best Practice Protocols for Physique Assessment in Sport. Singapore: Springer Singapore; 2018. pp. 87–99.

[129] Garr Barry V, Martin SL, Chandler-Laney P, Carter EB, Worthington CS. A Comparison of Bioimpedance Analysis vs. Dual X-ray Absorptiometry for Body Composition Assessment in Postpartum Women and Non-Postpartum Controls. Int J Environ Res Public Health. 2022;19(20):13636. 36294216 10.3390/ijerph192013636 PMC9602548

[130] Westerheim E, Øhman EA, Fossli M, Winkvist A, Henriksen HB, Brekke HK. Relative validity of bioelectrical impedance analysis in estimating body composition in women with overweight and obesity 2 weeks and 6 months postpartum. Food Nutr Res. 2025;69. 10.29219/fnr.v69.10869. 10.29219/fnr.v69.10869 PMC11836777 39974841

[131] Sun Y, Liu B, Snetselaar LG, Wallace RB, Caan BJ, Rohan TE, et al. Association of Normal-Weight Central Obesity With All-Cause and Cause-Specific Mortality Among Postmenopausal Women. JAMA Netw Open. 2019;2(7):e197337. 10.1001/jamanetworkopen.2019.7337. 31339542 10.1001/jamanetworkopen.2019.7337 PMC6659146

[132] Huai P, Liu J, Ye X, Li WQ. Association of Central Obesity With All Cause and Cause-Specific Mortality in US Adults: A Prospective Cohort Study. Front Cardiovasc Med. 2022;9:816144. 10.3389/fcvm.2022.816144. 35155634 10.3389/fcvm.2022.816144 PMC8832149

[133] Czernichow S, Kengne AP, Stamatakis E, Hamer M, Batty GD. Body mass index, waist circumference and waist-hip ratio: which is the better discriminator of cardiovascular disease mortality risk? evidence from an individual-participant meta-analysis of 82 864 participants from nine cohort studies. Obes Rev. 2011;12(9):680–7. 10.1111/j.1467-789X.2011.00879.x. 21521449 10.1111/j.1467-789X.2011.00879.x PMC4170776

[134] Wang L, Lee Y, Wu Y, Zhang X, Jin C, Huang Z, et al. A prospective study of waist circumference trajectories and incident cardiovascular disease in China: the Kailuan Cohort Study. Am J Clin Nutr. 2021;113(2):338–47. 10.1093/ajcn/nqaa331. 33330917 10.1093/ajcn/nqaa331

[135] Nevill AM, Duncan MJ, Myers T. BMI is dead; long live waist-circumference indices: But which index should we choose to predict cardio-metabolic risk? Nutr Metab Cardiovasc Dis. 2022;32(7):1642–50. 10.1016/j.numecd.2022.04.003. 35525679 10.1016/j.numecd.2022.04.003

[136] Long-Term Risk of Incident Type 2 Diabetes and Measures of Overall and Regional Obesity. The EPIC-InterAct Case-Cohort Study. PLos Med. 2012;9(6):e1001230. 10.1371/journal.pmed.1001230. 22679397 10.1371/journal.pmed.1001230 PMC3367997

[137] Ross R, Neeland IJ, Yamashita S, Shai I, Seidell J, Magni P, et al. Waist circumference as a vital sign in clinical practice: a Consensus Statement from the IAS and ICCR Working Group on Visceral Obesity. Nat Rev Endocrinol. 2020;16(3):177–89. 10.1038/s41574-019-0310-7. 32020062 10.1038/s41574-019-0310-7 PMC7027970

[138] Kimura Y, Inoue N, Yasunaga H. Body Roundness Index and Body Shape Index as Predictors for All-Cause Mortality Beyond Body Mass Index: Findings From a National Cohort Study. J Obes. 2026;2026:7923338. 10.1155/jobe/7923338. 41623543 10.1155/jobe/7923338 PMC12860135

[139] Minari TP, Uyemura JRR, Yugar-Toledo JC, Gouvea ECDP, Consenso-Martin LN, Pisani LP, et al. A body shape index and cardiometabolic risk markers: age- and sex-specific patterns. Clin Nutr ESPEN. 2026;73:103285. 10.1016/j.clnesp.2026.103285. 41967580 10.1016/j.clnesp.2026.103285

[140] Nikolic A, Mikovic Z, Gerginic V, Dragojevic Dikic S, Bojovic Jovic D, Salovic B, et al. Correlation of A Body Shape Index and Body Roundness Index as Novel Anthropometric Indices With Semen Analysis Parameters and IVF Outcomes. Am J Mens Health. 2025;19(5):15579883251380206. 10.1177/15579883251380206. 41025204 10.1177/15579883251380206 PMC12484924

