# Supplementary material for: Body composition changes in the postpartum: a systematic review on measurements and predictors
Source: BMC Pregnancy Childbirth. 2026 Jun 6;26:877. doi: 10.1186/s12884-026-09393-7 (PMC13459293; doi:10.1186/s12884-026-09393-7)
Supplement: Supplementary file 1 [file 12884_2026_9393_MOESM2_ESM.pdf]

Body composition changes in the postpartum: A systematic review on measurements and predictors

Journal- Current Obesity Reports

Malith Kumarasinghe[1], MD; Andrew P Hills[1], PhD; Kiran DK Ahuja[1], PhD

[1] School of Health Sciences, University of Tasmania, Launceston, Australia

Malith Kumarasinghe	**First Author**
	ORCID ID-0000-0001-8404-8623
	E-mail-Malith.Kumarasinghe@utas.edu.au
Andrew P Hills	ORCID ID-0000-0002-7787-7201
	E-mail-Andrew.Hills@utas.edu.au

Corresponding Author

Kiran DK Ahuja	ORCID ID-0000-0002-0323-4692
	E-mail-kiran.ahuja@utas.edu.au
	School of Health Sciences
	College of Health and Medicine
	University of Tasmania (UTAS)
	The Shed, 80, Cimitiere Street, Inveresk, Launceston, 7250, TAS Australia
	Phone +61 3 6324 5478

Supplementary Table 1: Eligibility criteria for the investigation of changes in postpartum body composition, and specific anthropometry and associated factors.

The table describes the inclusion and the exclusion criteria for selecting the published literature. Longitudinal observational studies with at least two measurements of maternal body composition and anthropometry in the postpartum period, excluding BMI or a single assessment during the postpartum period, if accompanied by a measurement at preconception, were included.

Supplementary Table 2: Key concepts for the investigation of changes in postpartum body composition and specific anthropometry and associated factors.

Two key concepts were chosen for the investigation of changes in postpartum body composition and specific anthropometry and associated factors, namely "Body composition" and "Postpartum". The key search terms were grouped under these two concepts either as free text terms or controlled vocabulary terms.

Supplementary Sheet 1: Search strategy

Six information sources were selected: Medline/PubMed, Scopus, EMBASE, CINAHL, Web of Science, and ProQuest. The search strings for each database are detailed.

Supplementary Figure 1: Flow of studies investigating the changes in postpartum body composition and specific anthropometry and associated factors.

Initially, 3,376 records were identified. After removing duplicates, 1,273 abstracts were screened. A total of 403 full-text articles were assessed for eligibility, and 86 studies were included in the final data extraction. Of these, 61 studies investigated

changes in postpartum body composition, while 16 each, examined the effects of breastfeeding and maternal risk factors on these changes.

Supplementary Figure 2: Change in postpartum fat mass (kg) based on the postpartum day of initial measurement and preconception BMI.

Graphs a1–a3 present findings from studies examining changes in maternal waist circumference (cm) during the postpartum period. Studies are grouped according to the timing of the initial fat mass assessment: within the first week after delivery, between the second and sixth weeks, and beyond the sixth week postpartum. Graphs b1–b3 and c1–c3 further stratify the studies by mean or median preconception BMI. As only one study included participants with a mean or median preconception BMI below 18.5 kg/m², this category is not shown.

Supplementary Figure 3: Change in postpartum waist circumference (cm) based on the postpartum day of initial measurement and preconception BMI.

Graphs a1–a3 summarise findings from studies that examined changes in maternal waist circumference (cm) during the postpartum period. The studies are grouped according to the timing of the initial fat mass assessment—within the first week after delivery, between the second and sixth weeks, or beyond the sixth week postpartum. Graphs b1–b3 and c1–c3 further categorise the studies based on the mean or median preconception BMI. Only one study included participants with a mean or median preconception BMI below 18.5 kg/m²; therefore, this category was not graphed.

Supplementary Figure 4: Association between HIV status and change in selected postpartum body composition and specific anthropometric indices.

The influence of HIV status on postpartum changes in fat mass ratio or percentage, fat mass (kg), fat-free mass (kg), and triceps skinfold thickness (mm) is illustrated. Dark and light colour shades indicate whether the associations are statistically significant or not, respectively. Blue represents lower body composition indices among HIV-positive postpartum mothers, whereas green denotes higher indices. The effects of HIV status on postpartum body composition are depicted up to two years after delivery.

Supplementary Table 1: Eligibility criteria for the investigation of changes in postpartum body composition, and specific anthropometry and associated factors.

**Criterion**	**Inclusion**
Population	Postpartum women
Exposure	All maternal and delivery-related factors that influence the change in body composition and specific anthropometry in the postpartum period including the 'time' factor
Comparison	Presence or absence of maternal/ delivery factor<br>OR<br>Between different levels of exposure to the factor based on the availability of data e.g., gestational age at delivery/ BMI at conception/ gestational weight gain/ anaemia<br>OR<br>No comparison
Outcome	Change in postpartum body composition:<br>At least two assessments of maternal body composition and specific anthropometry during the postpartum period<br>OR<br>A single assessment during the postpartum period if accompanied by a body composition and anthropometry assessment at preconception/ conception<br>AND<br>Any outcome measurement that is based on the fat mass or lean body mass, irrespective of the site
Time of data collection	The postpartum period from delivery up to two years
	**Exclusion**
Type of publication	Conference proceedings
Language (Main text)	Languages other than English
Study type	All intervention studies including randomised control trials
Outcome	Only BMI or weight
Time of publication	Published after 30th September 2025

Supplementary Table 2: Key concepts for the investigation of changes in postpartum body composition and specific anthropometry and associated factors.

	**Concept 1**	**Concept 2**
**Key concepts**	**Body composition**	**Postpartum**
**Free text terms / natural language terms**	Fat, muscle, bone, water	The period after delivery within one year
**Controlled vocabulary terms / subject terms**	Body composition, adiposity, fat mass, fat-mass, fat free mass, fat-free mass, lean mass, body fat, abdominal circumference, waist circumference	Postpartum, post-partum, postnatal, post-natal, interpregnancy, inter-pregnancy, inter pregnancy, interconception, inter-conception, inter conception

Supplementary Sheet 1

**PubMed**	
Search string	1. (postpartum period[MeSH Terms]) OR (postnatal care [MeSH Terms])<br>2. (postpartum[Title]) OR (post-partum[Title])<br>3. (postnatal[Title]) OR (post-natal[Title])<br>4. (interpregnancy[Title] OR "inter pregnancy"[Title:~0] OR inter-pregnancy[Title])<br>5. (interconception[Title] OR "inter conception"[Title:~0] OR inter-conception[Title])<br>6. #1 OR #2 OR #3 OR #4 OR #5<br>7. (body composition[MeSH Terms]) OR (Adiposity[Mesh Terms])<br>8. ("fat mass"[Title/Abstract:~0]) OR ("fat free mass"[Title/Abstract:~0] OR "fat-free mass"[Title/Abstract:~0] OR "lean mass" [Title/Abstract:~0])<br>9. ("body fat*" [Title/Abstract]) OR ("abdominal circumference" [Title/Abstract] :~0) OR ("waist circumference" [Title/Abstract:~0])<br>10. #7 OR #8 OR #9<br>11. (#6) AND (#10)<br>12. (animals [mh] NOT humans [mh])<br>13. #11 NOT #12

**Web of Science**	
Search string	TI=(postpartum)<br>TI=(postnatal)<br>#1 OR #2<br>TI=(interpregnancy) OR TI=(inter NEAR/0 pregnancy) OR TI=(inter-pregnancy)<br>#3 OR #4<br>TI=(interconception) OR TI=(inter NEAR/0 conception) OR TI=(inter-conception)<br>#5 OR #6<br>AB=(body NEAR/0 composition) OR TI=(adipos*) OR AB=(fat NEAR/0 mass)<br>AB=(fat NEAR/0 free NEAR/0 mass) OR AB=(fat-free NEAR/0 mass) OR AB=(lean NEAR/0 mass)<br>AB=(body NEAR/0 fat*) OR AB=(abdominal NEAR/0 circumference) OR AB=(waist NEAR/0 circumference)<br>#8 OR #9 OR #10<br>#7 AND #11<br>#7 AND #11 and 3.35 Zoology & Animal Ecology (Exclude – CitaABon Topics Meso)

**Scopus**	
Search string	TITLE (postpartum)<br>TITLE (postnatal)<br>#1 OR #2<br>TITLE (interpregnancy) OR TITLE (inter W/0 pregnancy) OR TITLE(inter-pregnancy)<br>#3 OR #4<br>TITLE (interconception) OR TITLE (inter W/0 conception) OR TITLE(inter-conception)<br>#5 OR #6<br>TITLE-ABS-KEY (body W/0 composition) OR TITLE-ABS-KEY (body W/0 fat*)<br>TITLE (adipos*)<br>TITLE-ABS-KEY (fat W/0 mass) OR TITLE-ABS-KEY (abdominal W/0 circumference) OR TITLE-ABS-KEY (waist W/0 circumference)<br>#8 OR #9 OR #10<br>TITLE-ABS-KEY (fat W/0 free W/0 mass) OR TITLE-ABS-KEY (fat-free W/0 mass) OR TITLE-ABS-KEY (lean W/0 mass)<br>#11 OR #12<br>SUBJAREA (vete)<br>#7 AND #13<br>#15 AND NOT #14

**Embase via Ovid**	
Search string	1. postpartum.m_titl.<br>2. postnatal.m_titl.<br>3. 1 or 2<br>4. interpregnancy.m_titl.<br>5. inter pregnancy.m_titl.<br>6. inter-pregnancy.m_titl.<br>7. 4 or 5 or 6<br>8. 3 or 7<br>9. interconception.m_titl.<br>10. inter conception.m_titl.<br>11. inter-conception.m_titl.<br>12. 9 or 10 or 11<br>13. 8 or 12<br>14. exp body composition/<br>15. exp body fat distribution/ or exp body fat/<br>16. adipos*.m_titl.<br>17. exp fat mass/<br>18. 14 or 15 or 16 or 17<br>19. exp fat free mass/<br>20. exp lean body weight/<br>21. exp waist circumference/<br>22. exp abdominal circumference/<br>23. 19 or 20 or 21 or 22<br>24. 18 or 23<br>25. 13 and 24

**CINAHL**	
Search string	1. TI postpartum OR TI postnatal<br>2. TI interpregnancy OR TI inter pregnancy OR TI inter-pregnancy<br>3. TI interconception OR TI inter conception OR TI inter-conception<br>4. (S1) OR (S2) OR (S3)<br>5. AB body composition OR AB body fat* OR TI adipos*<br>6. AB fat mass OR AB fat free mass OR AB fat-free mass OR AB lean mass<br>7. AB waist circumference OR AB abdominal circumference<br>8. (S5) OR (S6) OR (S7)<br>9. (S4) AND (S8)

**ProQuest**	
Search string	1. title(postpartum) OR title(post-partum)<br>Limited by:<br>Source type:Conference Papers & Proceedings, Dissertations & Theses<br>Document type:Conference Paper, Conference Proceeding, Dissertation/Thesis<br>2. title(postnatal) OR title(post-natal)<br>Limited by:<br>Source type:Conference Papers & Proceedings, Dissertations & Theses<br>Document type:Conference Paper, Conference Proceeding, Dissertation/Thesis<br>3. title(interpregnancy) OR title(inter-pregnancy) OR title("inter pregnancy")<br>Limited by:<br>Source type:Conference Papers & Proceedings, Dissertations & Theses<br>Document type:Conference Paper, Conference Proceeding, Dissertation/Thesis<br>4. title(interconception) OR title(inter-conception) OR title("inter conception")<br>Limited by:<br>Source type:Conference Papers & Proceedings, Dissertations & Theses<br>Document type:Conference Paper, Conference Proceeding, Dissertation/Thesis<br>5. [S1] OR [S2] OR [S3] OR [S4]<br>6. abstract("body composition") OR title(adipos*) OR abstract("fat mass")<br>Limited by:<br>Source type:Conference Papers & Proceedings, Dissertations & Theses<br>Document type:Conference Paper, Conference Proceeding, Dissertation/Thesis<br>7. abstract("fat free mass") OR abstract("fat-free mass") OR abstract("lean mass")<br>Limited by:<br>Source type:Conference Papers & Proceedings, Dissertations & Theses<br>Document type:Conference Paper, Conference Proceeding, Dissertation/Thesis<br>8. abstract(body fat*)<br>Limited by:<br>Source type:Conference Papers & Proceedings, Dissertations & Theses<br>Document type:Conference Paper, Conference Proceeding, Dissertation/Thesis<br>9. abstract("waist circumference") OR abstract("abdominal circumference")<br>Limited by:<br>Source type:Conference Papers & Proceedings, Dissertations & Theses<br>Document type:Conference Paper, Conference Proceeding, Dissertation/Thesis<br>10. [S6] OR [S7] OR [S8] OR [S9]<br>11. [S5] AND [S10]

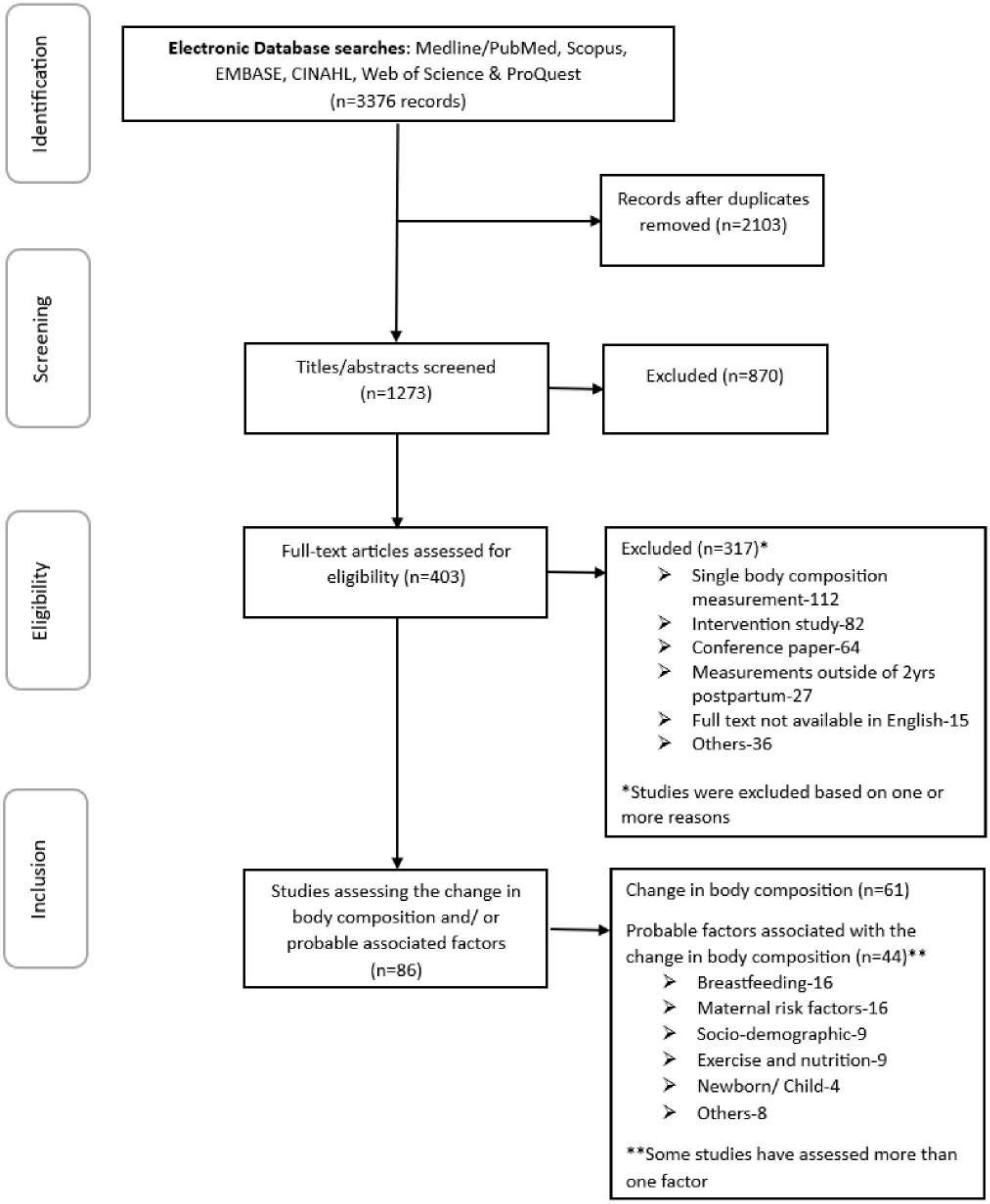

Supplementary Figure 1: Flow of studies investigating the changes in postpartum body composition and specific anthropometry and associated factors.

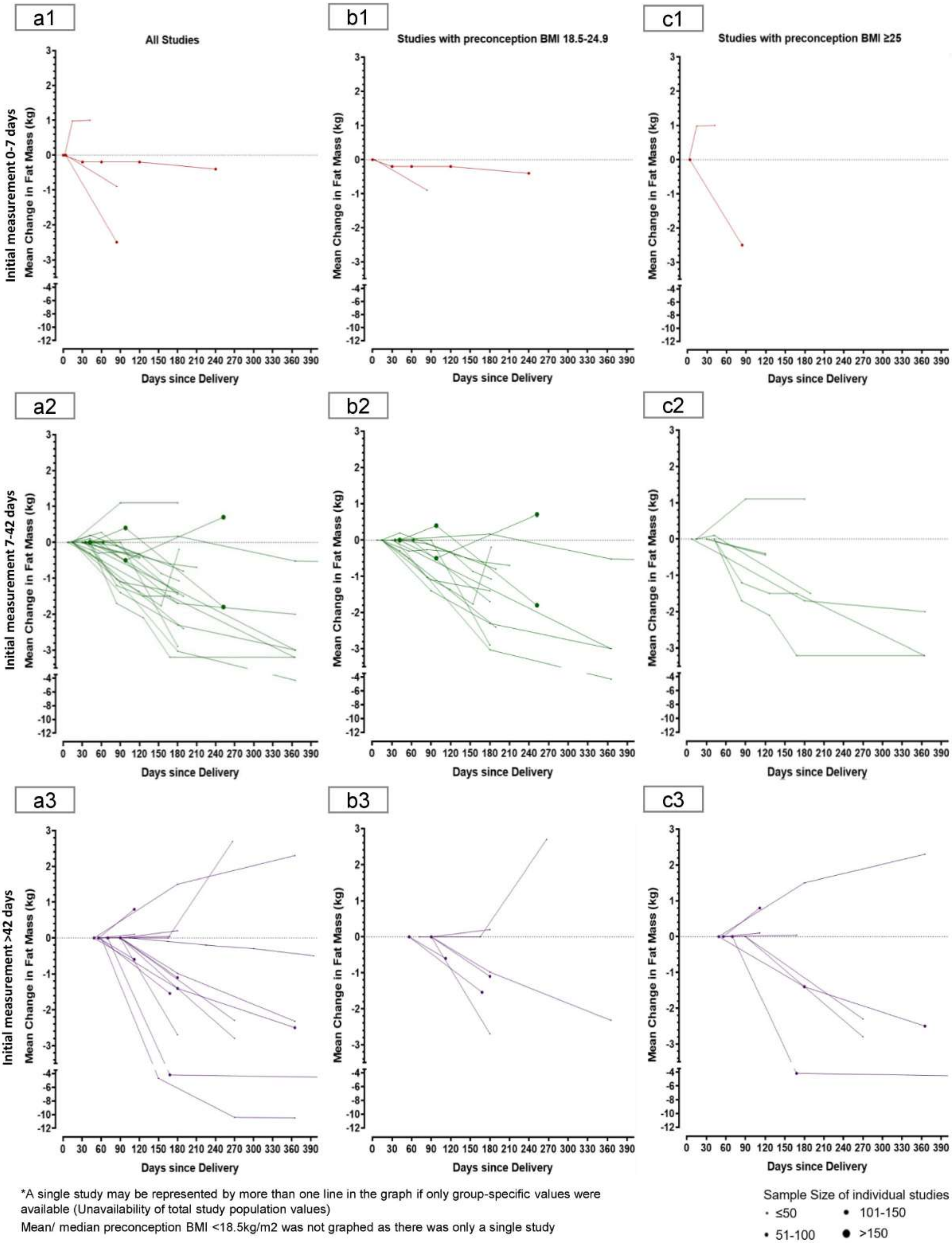

Supplementary Figure 2 Change in postpartum fat mass (kg) based on the postpartum day of initial measurement and preconception BMI.

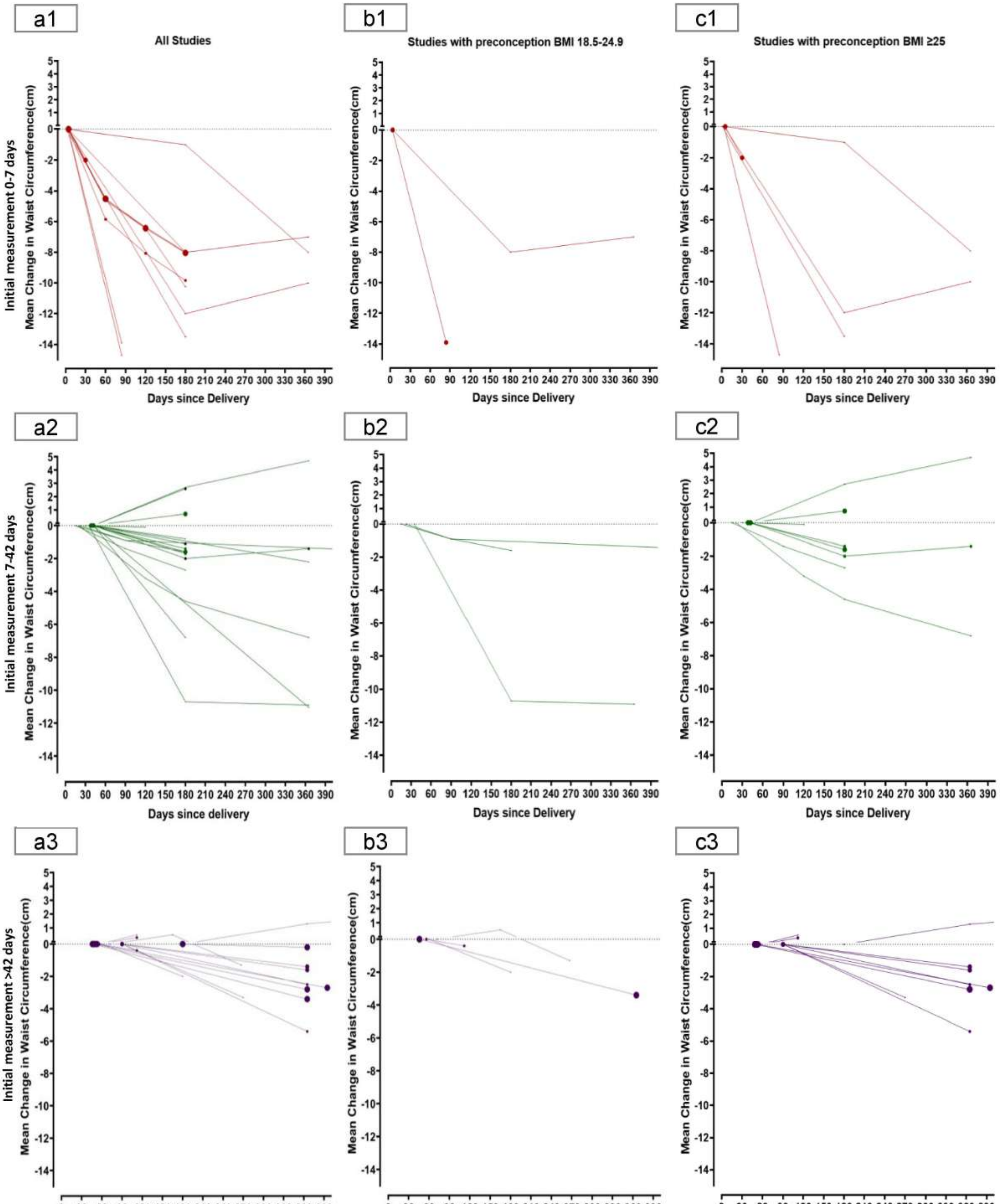

*A single study may be represented by more than one line in the graph if only group-specific values were available (Unavailability of total study population values)

Mean/ median preconception BMI <18.5kg/m2 was not graphed as there was only a single study

Sample Size of individual studies
- ≤50
- 51-100
- 101-150
- >150

Supplementary Figure 3 Change in postpartum waist circumference (cm) based on the postpartum day of initial measurement and preconception BMI.

Indicator	Study	Groups	Sample size*	Technique	≤7D	8-14D	15-30D	31-42D	43-90D	91-180D	181-270D	271-365D	366-730D
**Fat Mass Ratio/ Percentage**	Papathakis 2006	HIV negative[a]: HIV positive	127-113	BIA					Lower (non-significant)	Lower (non-significant)			
**Fat Mass (kg)**	Widen 2019	HIV negative[a]: HIV positive	264-236	BIA				Lower (non-significant)		Lower (significant)	Higher (non-significant)		
	Widen 2017	HIV negative[a]: HIV positive	246-243	BIA	Higher (non-significant)	Higher (non-significant)	Higher (non-significant)	Higher (non-significant)	Higher (non-significant)	Higher (non-significant)	Higher (non-significant)	Higher (non-significant)	
	Papathakis 2006	HIV negative[a]: HIV positive	127-113	BIA					Lower (non-significant)	Lower (non-significant)			
**Fat Free Mass (kg)**	Widen 2019	HIV negative[a]: HIV positive	264-236	BIA				Lower (significant)		Lower (significant)	Lower (significant)		
	Widen 2017	HIV negative[a]: HIV positive	246-243	BIA	Lower (non-significant)	Lower (non-significant)	Lower (non-significant)	Lower (non-significant)	Lower (non-significant)	Lower (non-significant)	Lower (non-significant)	Lower (non-significant)	
	Papathakis 2006	HIV negative[a]: HIV positive	127-113	BIA					Higher (non-significant)	Higher (non-significant)			
**Triceps Skinfold Thickness (mm)**	Widen 2019	HIV negative[a]: HIV positive	264-236	N/A				Lower (significant)		Lower (significant)	Lower (significant)		
	Papathakis 2006	HIV negative[a]: HIV positive	127-113	N/A					Lower (significant)	Lower (significant)			

Lower body composition indices (significant) between one or more time points compared to reference group

Lower body composition indices (non-significant) between two time points compared to reference group

Higher body composition indices (significant) between one or more time points compared to reference group

Higher body composition indices (non-significant) between one or more time points compared to reference group

No significant difference/ association between the change in body composition indices compared to reference group (Direction cannot be determined)

*Intial measurement-final measurement (If positive for loss to follow up)

[a]Reference group

D-Days since delivery

BIA-Bioelectrical impedance analysis

Supplementary Figure 4: Association between HIV status and change in selected postpartum body composition and specific anthropometric indices.
